# Transcriptional coordination between intergenic RNA polymerase II-bound regions and nearby genes reveals functional specialization and disease associations in peripheral blood

**DOI:** 10.1016/j.csbj.2025.11.060

**Published:** 2025-11-28

**Authors:** Vijaykumar Yogesh Muley, Andrée Delahaye-Duriez

**Affiliations:** a Université Paris Cité, Inserm, NeuroDiderot, Paris F-75019, France; b UFR de Santé, Médecine et Biologie Humaine, Université Sorbonne Paris Nord, Bobigny F-93000, France; c Unité Fonctionnelle de Médecine Génomique et Génétique Clinique, Hôpital Jean Verdier, AP-HP, Bondy F-93140, France

**Keywords:** RNA Polymerase II, Transcription, Gene expression, Non-coding RNA, Cis-regulatory elements, Distal regulatory elements, RNA-Seq, Intergenic transcription, Next-generation sequencing, Enhancer RNAs, Enhancer

## Abstract

Recent studies have shown that RNA polymerase II (RNAPII) frequently occupies regions outside conventional promoters and gene bodies, forming intergenic RNAPII-bound regions (iRNAPII-BRs) that are actively transcribed and involved in gene regulation. We assessed the transcriptional activity of 181,547 iRNAPII-BRs from a genome-wide atlas and their impact on the expression of nearby gene in peripheral blood. The iRNAPII-BRs were often associated with structurally complex longer genes located in gene deserts. Their strongest transcriptional activity peaks occurred within 10 kb upstream from transcription start sites (TSSs), indicating a close regulatory link. Genes were grouped into six categories according to their transcriptional coordination with the nearest iRNAPII-BRs. Housekeeping genes tended to be transcribed independently, whereas blood tissue-specific genes were highly coordinated with iRNAPII-BR transcription. Genes with neuronal functions were frequently transcribed without active iRNAPII-BRs, suggesting basal promoter-driven expression with potential regulation by iRNAPII-BRs in response to blood-brain cross-talk cues. We found that 4507 iRNAPII-BRs were positively correlated with gene expression, whereas 793 were negatively correlated with gene expression implying a possible role in repression. The presence of transcription factor binding sites common to iRNAPII-BRs and promoters suggests a cooperative regulatory mechanism. Finally, we observed a differential expression of iRNAPII-BRs and nearby genes—*ADRB2*, *CXCL8*, *SFN*, and *GPR3*—in major depressive disorder, indicating that iRNAPII-BR-associated transcription may contribute to the pathophysiology of this disorder.

## Graphical Abstract

**Figure:**
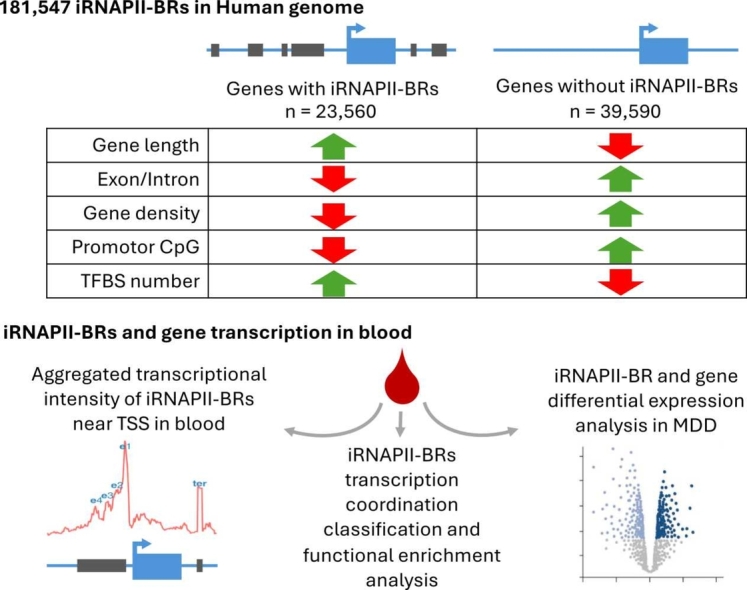

## 1 Introduction

RNA polymerases (RNAPs) are multi-subunit enzymes that transcribe DNA into RNA. Eukaryotes possess three distinct RNAPs. RNAPII primarily transcribes protein-coding genes, whereas RNAPI and RNAPIII synthesize non-coding RNAs (ncRNAs) [21]. Transcription begins when RNAPII is recruited to promoter regions by sequence-specific transcription factors (TFs) that guide it to the transcription start site (TSS) [18]. Promoters and other cis-regulatory elements (CREs)—including enhancers, silencers, and insulators—control the timing and magnitude of gene expression [45]. Promoters typically reside within ∼1 kb upstream from the TSS, whereas distal enhancers regulate transcription from up to 1 Mb away from the TSS, through the recruitment of coactivators and chromatin remodelers to promote RNAPII binding and enhancer–promoter looping. These long-range enhancer–promoter interactions are also RNAPII-dependent [70].

Promoter-associated gene transcription has been studied in detail [18]. RNAPII commonly initiates divergent (bidirectional) transcription at protein-encoding gene promoters [57], generating a full length downstream mRNA, and short, highly unstable polyadenylated promoter upstream transcripts (PROMPTs) [52]. RNAPII also binds and transcribes intergenic regions far from annotated genes [6]. We refer to these regions hereafter as intergenic RNAPII-bound regions (iRNAPII-BRs). Genome-wide studies have revealed the existence of thousands of actively transcribed iRNAPII-BRs (tiRNAPII-BRs) [33], [54], producing transcripts of various lengths, strand specificities, directionalities, and polyadenylation levels [4]. Some of these transcripts may arise from previously unannotated genes, but most originate from enhancers and form a distinct class of ncRNAs known as enhancer RNAs (eRNAs) [33], [54].

The function of transcripts from iRNAPII-BRs or enhancers remains unclear [50]. Some may represent divergent transcription noise from enhancer–promoter loops [65], whereas others may promote the formation or maintenance of open chromatin states [42], [47]. Furthermore, the eRNA itself or its transcription may have regulatory functions [33].

A probabilistic model (Tfit) of RNAPII loading, initiation, and elongation assuming unified genome-wide behavior accurately predicts bidirectionally transcribed CREs from nascent transcription data [7]. These regions precisely overlap regulatory chromatin marks and align with active transcription-regulating elements identified by global run-on sequencing (GRO-seq) by the discriminative regulatory element detection method (dREG) [17]. Thus, iRNAPII-BRs and their transcriptional activity provides an elegant solution to define and interpret regulatory elements influencing the expression of nearby genes.

de Langen et. al. analyzed over 1000 RNAPII ChIP-seq datasets from diverse tissues, cells, and cancers [35]. They generated a consensus map of 181,547 iRNAPII-BRs, excluding genic regions, which were defined as extending from 1 kb upstream from the TSS to 1 kb downstream from the transcription termination site.

In this study, we analyzed the genomic context of this set of 181,547 iRNAPII-BRs and nearest genes. Genes located close to iRNAPII-BRs were typically found in regions with a low gene density and had complex structures. We detected transcriptional activity at 58,259 iRNAPII-BRs in peripheral blood. We subsequently classified the nearest genes based on their transcriptional status and their dependence on both the presence of iRNAPII-BRs and their transcriptional activity, revealing their functional consequences.

## 2 Results

Our dataset contained the genomic coordinates of 181,170 iRNAPII-BRs from the canonical chromosomes (Dataset S1), derived from the 181,547 iRNAPII-BRs identified in the study by [35].

We compared the iRNAPII-BRs with CRE catalogs from DBNascent, ENCODE, FANTOM5, EnhancerAtlas2.0, and NET-CAGE databases [1], [16], [24], [28], [58]. DBNascent compiles bidirectionally transcribed intergenic regions detected in nascent transcription assays [58]. ENCODE defines CREs on the basis of histone modifications, DNase hypersensitivity, and CTCF-binding profiles [1]. FANTOM5 uses principally CAGE data to identify transcribed enhancers [3], whereas NET-CAGE maps active enhancers from the 5′ ends of nascent RNAs independent of RNA stability [28]. EnhancerAtlas2.0 integrates multiple high-throughput datasets, including nascent RNA and RNAPII ChIP-seq, to produce comprehensive enhancer annotations [24].

The iRNAPII-BRs overlapped strongly with all enhancer datasets (Fisher’s exact test, p < 2 × 10⁻¹⁶). Specifically, 91.95 % overlapped with EnhancerAtlas2.0, 69.30 % with DBNascent, and 65.03 % with ENCODE, whereas overlap rates with FANTOM5 enhancers was lower (≤16.44 %) (Table 1). The broader overlap with EnhancerAtlas2.0 probably reflects the use of wider genomic intervals in this dataset, consistent with observations from the DBNascent study [58]. In DBNascent, the annotations generated by dREG and Tfit displayed a high degree of concordance with iRNAPII-BRs, with a greater overlap for dREG than for Tfit (Figure S1), reflecting the methodological differences between these two approaches [17], [7]. The iRNAPII-BRs overlapped strongly with both distal and proximal enhancer-like elements in ENCODE, but displayed a minimal overlap with CTCF-only, DNase–H3K4me3, and promoter-like regions (Figure S1).

**Table 1:** Overlap of iRNAPII-BRs with *cis*-regulatory elements (CREs) from multiple datasets.

**Dataset**	**Total number of CREs in dataset**	**CREs overlapping with iRNAPII-BRs**	**Overlapping iRNAPII-BRs**	**Coverage of iRNAPII-BRs (%)**
**DBNascent**	847521	158102	125548	69.30
**Fantom5**	63285	24579	22460	12.40
**Encode**	926535	206421	117815	65.03
**EnhancerAtlas2.0**	238092	167909	166584	91.95
**NET-CAGE**	86000	33718	29792	16.44

Overall, 98.97 % of iRNAPII-BRs overlapped with at least one enhancer dataset. Even after excluding the broader EnhancerAtlas2.0 regions, 82.79 % were supported by other resources, providing additional support for their identity as enhancers, this value being higher than the 66 % reported for enhancer-like classification by [35]. The strong concordance with DBNascent provides additional support for a bidirectional transcriptional activity of iRNAPII-BRs.

### 2.1 Comparison of genes with and without iRNAPII-BR linkage

Despite extensive efforts to predict and characterize enhancers, the features potentially distinguishing between genes with and without linked enhancers, such as gene length, exon/intron ratio, and genomic context, remain poorly explored.

We investigated the patterns distinguishing between genes associated and not associated with iRNAPII-BRs by mapping iRNAPII-BR coordinates to 63,150 genes on canonical chromosomes annotated in the ENSEMBL GRCh38 genome assembly [27]. Each iRNAPII-BR can potentially regulate multiple genes, and conversely, a single gene may be influenced by several iRNAPII-BRs [63]. Various strategies can be used to associate each iRNAPII-BR with its potential target genes [3], [58], [63]. Here, we linked each iRNAPII-BR to the nearest gene on the same chromosome [75]. This approach is simple, transparent, and avoids arbitrary assumptions about fixed distance thresholds from the TSS (e.g., 1 kb–1 Mb) or the inclusion of multiple nearest genes (k-nearest). Moreover, the nearest-gene strategy assumes that an iRNAPII-BR probably exerts its primary regulatory effect on the closest gene. We calculated the distance between the center of each iRNAPII-BR and the TSS of the gene nearest to it. The iRNAPII-BR farthest upstream was located 476,417 bases away from the nearest gene, whereas the farthest downstream was located 553,661 bases away from the nearest gene (Dataset S1). These distances fall within the ∼1 Mb range commonly considered to be associated with enhancer-mediated gene regulation [45].

**Fig. 1:**
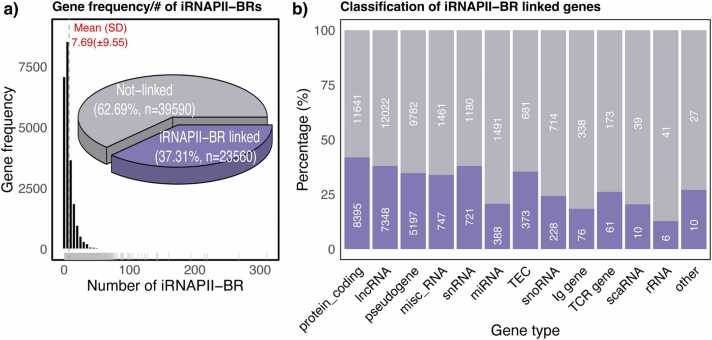
Distribution and classes of genes with and without associated iRNAPII-BR.

We found that, 23,560 (37.31 %) of the 63,150 genes were linked to one or more iRNAPII-BRs (Fig. 1a). These genes were associated with a mean of 7.69 (SD =±9.55) iRNAPII-BRs, and were potentially regulated by iRNAPII-BRs, promoters, or both. The remaining 62.69 % of the genes were not associated with any iRNAPII-BR. They may be regulated solely by promoter-based mechanisms and/or by RNAPII-independent regulation. We found that 41.63 % (8395) of protein-coding genes (20,036 in total) were linked to at least one iRNAPII-BRs (Fig. 1b). By contrast, most small ncRNAs, such as miRNAs, snoRNAs, and rRNAs, were less likely to be linked to iRNAPII-BRs. This finding is consistent with the general tendency of protein-coding genes to be transcribed by RNAPII, whereas ncRNAs are more likely to be transcribed by RNAPI or RNAPIII [15].

**Fig. 2:**
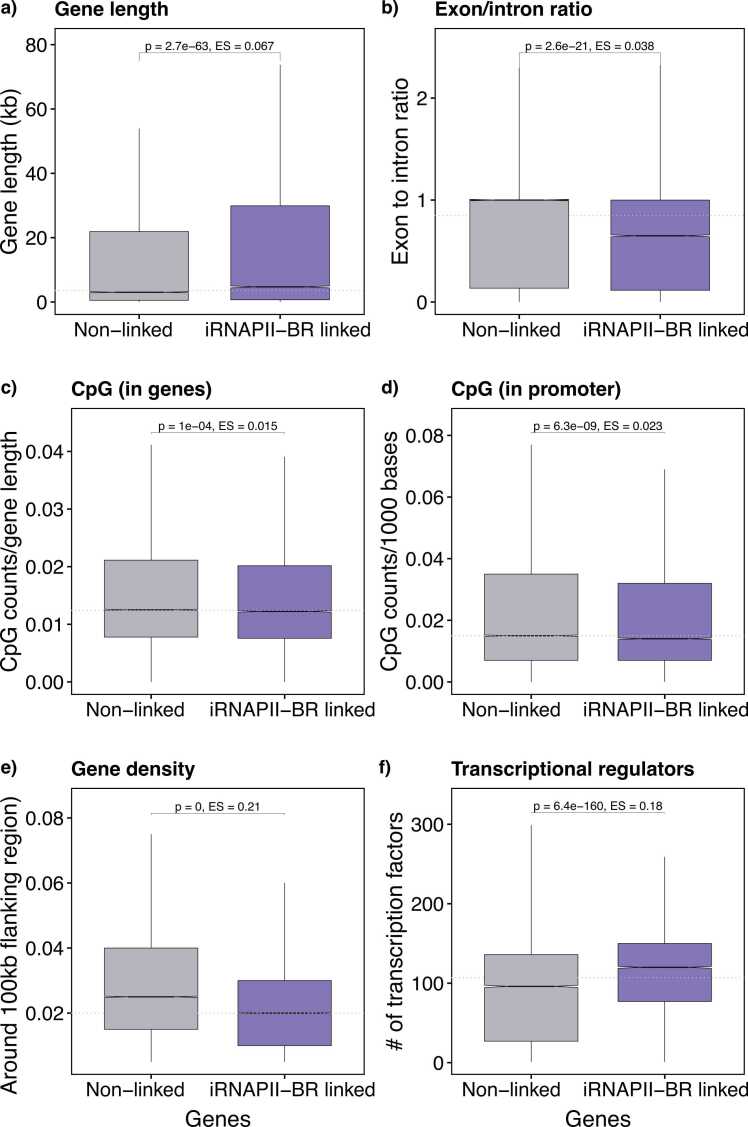
Characteristic features of genes with (in purple n = 23,560) and without associated iRNAPII-BR (in gray n = 39,590). The p-value (p) and effect size (ES) for comparisons between two groups were determined in Wilcoxon rank-sum tests.

The genes linked to iRNAPII-BRs had distinctive genomic features (Fig. 2). They tended to be significantly longer (Fig. 2a) and had a lower exon-to-intron ratio (Fig. 2b), indicating a higher prevalence of large introns and/or larger number of introns. These features are consistent with reported findings that longer genes are more likely to undergo alternative splicing [26]. A lower exon-to-intron ratio also suggests greater potential for alternative splicing, allowing multiple isoforms to be produced from the same gene, with different functions in different tissues. Moreover, tissue-specific or stimulus-induced genes tend to be longer than housekeeping genes [74], and are often associated with active enhancers [34]. Both gene length and splicing complexity affect transcriptional timing and efficiency, as longer transcripts and complex pre-mRNA processing require more time [11]. Genes with multiple promoters have been shown to harbor more enhancers, with enhancer diversity contributing to the selective expression of isoforms during T-cell differentiation [44]. Thus, iRNAPII-BRs may provide additional regulatory input for optimizing the expression of long, structurally complex and isolated genes on chromosomes.

Genes linked to iRNAPII-BRs had slightly lower CpG content within the body of the gene than unlinked genes (Fig. 2c), but significantly fewer within their promoters (defined as lying in the region 1 kb upstream of the TSS) (Fig. 2d). Human promoters naturally segregate into high- and low-CpG classes, with ∼72 % of genes having high-CpG promoters and ∼28 % having low-CpG promoters [55]. High-CpG promoters are typically found in housekeeping genes, whereas tissue-specific genes are more likely to have low-CpG promoters [36], [74]. Our results are consistent with this, as iRNAPII-BR-linked genes appear to have low-CpG promoters.

We calculated gene density in the 100 kb region flanking genes with and without iRNAPII-BR linkage. The iRNAPII-BR-linked genes were located in regions of significantly lower gene density (Fig. 2e). This trend was confirmed by a scatter plot of the relationship between the number of iRNAPII-BRs around each gene and local gene density within a window of ±100 kb (Figure S2). Following the binning of data according to gene density, an even stronger negative correlation was observed between mean iRNAPII-BR count and surrounding gene density (Figure S2). These results are consistent with the tendency of genes with spatially or temporally restricted expression to be located in “gene deserts”, which is thought to help maintain regulatory specificity between enhancers and their target promoters [64]. For instance, when the β-globin locus control region (LCR) is inserted into a gene-rich domain, it ectopically activates nearby genes, disrupting normal specificity [51].

We investigated the regulatory landscape by assigning transcriptional regulators to each gene via a human regulatory network of TFs compiled from 14 data sources [49]. Genes without iRNAPII-BRs were regulated by around 90 TFs. By contrast, iRNAPII-BR-linked genes were associated with more than 120 TFs (Fig. 2f). The promoter-based initiation of transcription typically involves ∼90 transcription regulatory factors, including RNAPII [39]. The larger number of TFs linked to iRNAPII-BR-associated genes suggests additional regulatory complexity involving the iRNAPII-BRs themselves.

We also observed functional differences between the two gene groups (Figure S3). The genes linked to iRNAPII-BRs displayed an enrichment in processes such as cell adhesion, signaling, and development. By contrast, genes without iRNAPII-BRs displayed an enrichment in genes for the sensory perception of smell and for post-transcriptional regulatory processes. However, these results may be biased towards protein-coding genes, which are better annotated than ncRNA genes.

To exclude the possibility that gene biotype influenced our findings, we repeated all analyses using only protein-coding genes, comparing those linked and unlinked to iRNAPII-BRs. The patterns were broadly consistent with the genome-wide results (Figure S4), except for CpG content within the gene body (Figure S4), which did not show significant difference between iRNAPII-BR-linked and -unlinked protein-coding genes (Fig. 2). These results suggest that the gene CpG-related differences seen in the full dataset may have been driven primarily by non–protein-coding biotypes (such as lncRNAs or pseudogenes).

Overall, our findings indicate that genes linked to iRNAPII-BRs exhibit more complex architectures, greater transcript lengths, low-CpG promoters, and are typically located in gene-poor regions. They are also regulated by a higher number of transcription factors, reflecting a broader regulatory input. Together, these features are hallmarks of tissue-specific genes.

### 2.2 Genome-wide transcriptional landscape of iRNAPII-BRs and genes in peripheral blood

We analyzed transcriptionally active iRNAPII-BRs in peripheral blood using in-house RNA-Seq data [46]. The dataset comprised 87 samples, including 38 from females with major depressive disorder (MDD) and 49 from healthy controls. Reads were aligned with the ENSEMBL human genome assembly to 1 kb regions centered on 181,170 iRNAPII-BRs and to the bodies of 63,150 ENSEMBL genes [27], [40]. Read count matrices were generated separately for genes and iRNAPII-BRs [41]. Outlier samples with weak correlations were excluded (Figure S5). We retained iRNAPII-BRs and genes with at least one read in three samples (Dataset S2 and S3).

**Fig. 3:**
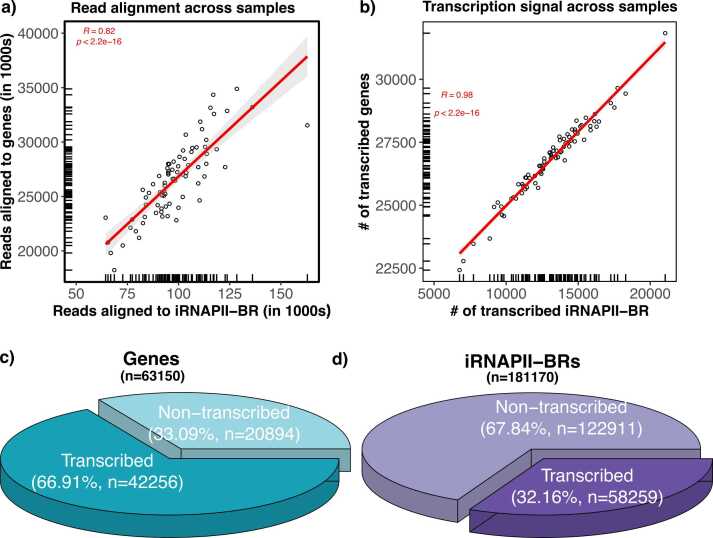
Comparative analysis of transcriptional activity at iRNAPII-BR and genes across samples. Panel a) presents the correlation between reads mapped to genes and iRNAPII-BRs across samples. Panel b) shows the correlation between the number of transcribed genes and iRNAPII-BRs across samples. Panels c) and d) show the overall distribution of transcribed and non-transcribed genes and iRNAPII-BRs. A gene or iRNAPII-BR was considered transcribed if one read was observed in at least three samples.

Overall, ∼97.65 % of sequenced reads mapped to the genome, with 82.19 % of these reads mapping to genes and only 0.25 % to iRNAPII-BRs. It should be noted that our samples were enriched in polyadenylated (poly(A)) RNA species and therefore contained primarily mRNAs. By contrast, most transcripts from intergenic regions are short-lived, non-polyadenylated, and rapidly degraded by the RNA exosome [52], probably accounting for the much smaller number of reads mapping to iRNAPII-BRs. Nevertheless, gene and iRNAPII-BR read counts were strongly correlated across samples (R = 0.82, p < 2.2e-16) (Fig. 3a), indicating a strong concordance between transcriptional activity and regulatory element engagement, and reflecting robust biological signal. Likewise, in individual samples, a mean of 26,834.47 genes (SD = 1529.86) and 13,147.78 iRNAPII-BRs (SD = 2549.13) were transcribed. These counts were also highly correlated across samples (R = 0.98, p < 2.2e-16) (Fig. 3b).

A transcriptional signal was detected for 66.91 % (n = 42,256) of genes and 32.16 % (n = 58,259) of iRNAPII-BRs (Fig. 3c,d). The 58,259 tiRNAPII-BRs were associated with 17,456 genes, 12,649 of which were cotranscribed with iRNAPII-BRs (72.46 %), whereas 4807 were not (27.54 %).

Overall, we observed an almost perfect positive correlation between the number of genes and iRNAPII-BRs transcribed across samples. This relationship indicates that a higher abundance of iRNAPII-BR transcripts is associated with a greater likelihood of gene transcription, consistent with a role for iRNAPII-BRs analogous to that of enhancers in gene activation. Moreover, bulk poly(A)-enriched RNA-seq data may still capture a modest but biologically meaningful fraction of iRNAPII-BR transcripts.

### 2.3 Functional consequences of iRNAPII-BR and nearest gene transcription

**Fig. 4:**
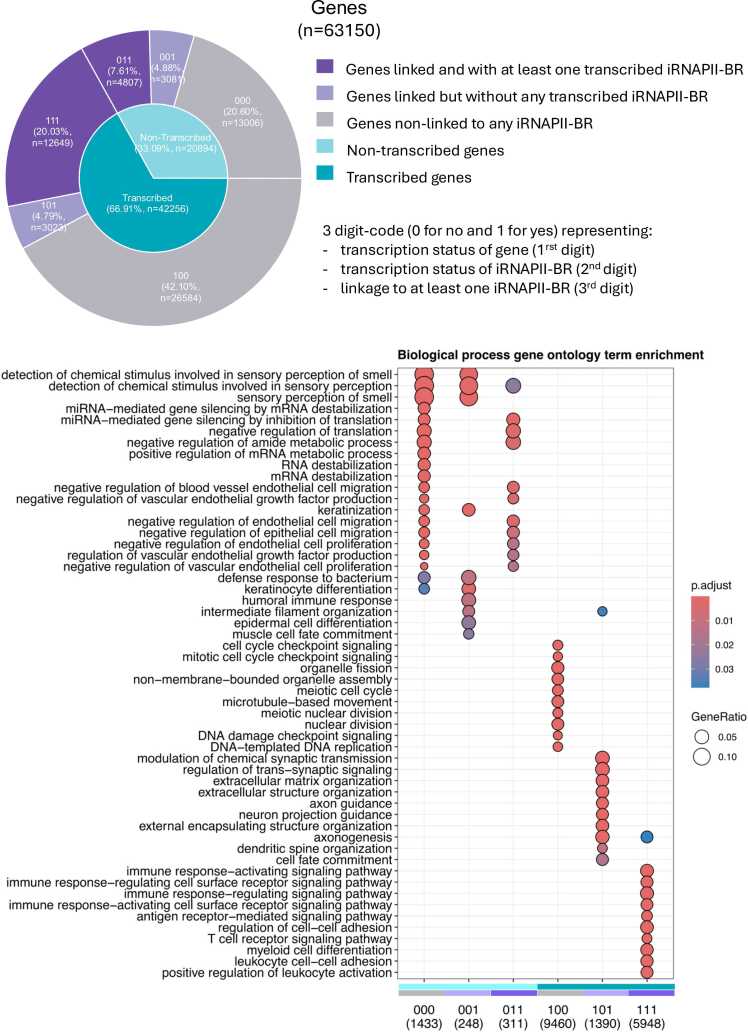
Gene ontology enrichment analysis of six gene groups defined on the basis of three binary statuses concerning gene transcription, iRNAPII-BR transcription and whether the gene was linked to an iRNAPII-BR. The first digit of the group name indicates gene transcription status (1 = transcribed, 0 = not transcribed), the second digit indicates iRNAPII-BR transcription status (1 = transcribed, 0 = not transcribed), and the third digit indicates whether the gene is linked to an iRNAPII-BR (1 = linked, 0 = not linked). This classification assigned genes to six groups based on these binary combinations.

We assessed the functional role of iRNAPII-BR transcription by classifying genes on the basis of three binary features: gene transcription status (1 = transcribed, 0 = not transcribed), iRNAPII-BR transcription status (1 = transcribed, 0 = not transcribed), and whether the gene was linked to an iRNAPII-BR (1 = linked, 0 = not linked). This classification resulted in six gene groups (Fig. 4). Gene Ontology (GO) enrichment analysis showed that each group was enriched in a different set of biological functions (Fig. 4).

Group 000 (20.60 % not transcribed genes, unlinked to iRNAPII-BR) was enriched in terms relating to the inhibition of epithelial/endothelial proliferation and migration, amide metabolism, RNA destabilization, miRNA-mediated silencing, and sensory perception. Group 001 (4.88 % not transcribed genes, linked to non-transcribed iRNAPII-BRs) displayed enrichment in sensory perception, keratinization, and muscle cell fate—functions. Group 011 (7.62 % not transcribed genes, linked to tiRNAPII-BR) was enriched in GO terms overlapping with those of Group 000 and included genes involved in translation repression and the inhibition of epithelial/endothelial processes. Group 011 genes were either transcriptionally paused or repressed by the closest transcribed iRNAPII-BRs. eRNAs can form ERα-centered complexes with KDM2A to remove RNAPII from enhancers and suppress target gene transcription [66]. These groups appeared to contain genes encoding proteins with functions predominantly unrelated to blood.

Group 100 (42.10 % transcribed genes, unlinked to iRNAPII-BR) was the largest group. The genes in this group were enriched in housekeeping functions such as DNA damage response, mitotic/meiotic checkpoints, organelle fission, and microtubule dynamics. These genes are probably regulated by promoters and RNAPII-independent mechanisms.

Group 101 (4.79 % transcribed genes, linked to non-tiRNAPII-BR) displayed an enrichment in neural processes, including axon guidance, synapse organization, and extracellular matrix remodeling. Interestingly, these genes are transcribed, whereas the iRNAPII-BRs closest to them are not. We speculate that their basal transcription is primarily promoter-driven, but they may gain additional regulatory input from the iRNAPII-BR transcription, specifically from the brain. Such genes could be potential candidates for blood–brain communication.

Group 111 (20.03 % transcribed genes linked to tiRNAPII-BRs) was enriched in immune and hematopoietic functions, including leukocyte differentiation, immune cell activation, and antigen receptor signaling, suggesting a tissue-specific regulatory role. These observations are consistent with the functions of iRNAPII-BRs being similar to that of enhancers in defining tissue/cell type specific gene expression programs [3]

In summary, transcribed genes not associated with any iRNAPII-BR tend to perform housekeeping functions. By contrast, genes co-transcribed with iRNAPII-BRs tend to have specialized, tissue-specific roles, consistent with an enhancer-like functions of iRNAPII-BRs. Notably, the transcribed genes associated with untranscribed iRNAPII-BRs (Group 101) are enriched in neural functions and may potentially be involved in the regulatory crosstalk between blood and brain.

### 2.4 Spatial genomic distribution and transcript abundance of iRNAPII-BRs

We investigated the spatial distribution of tiRNAPII-BR transcription relative to gene TSSs by assigning mean read counts normalized across samples to each tiRNAPII-BR [43]. We then defined a ± 20 kb window around each TSS, which we split into 147 bins of equal-size to approximate nucleosome-scale resolution. For each bin, we summed the normalized read counts of the tiRNAPII-BRs overlapping the bin concerned to create an aggregated transcriptional intensity profile [75].

**Fig. 5:**
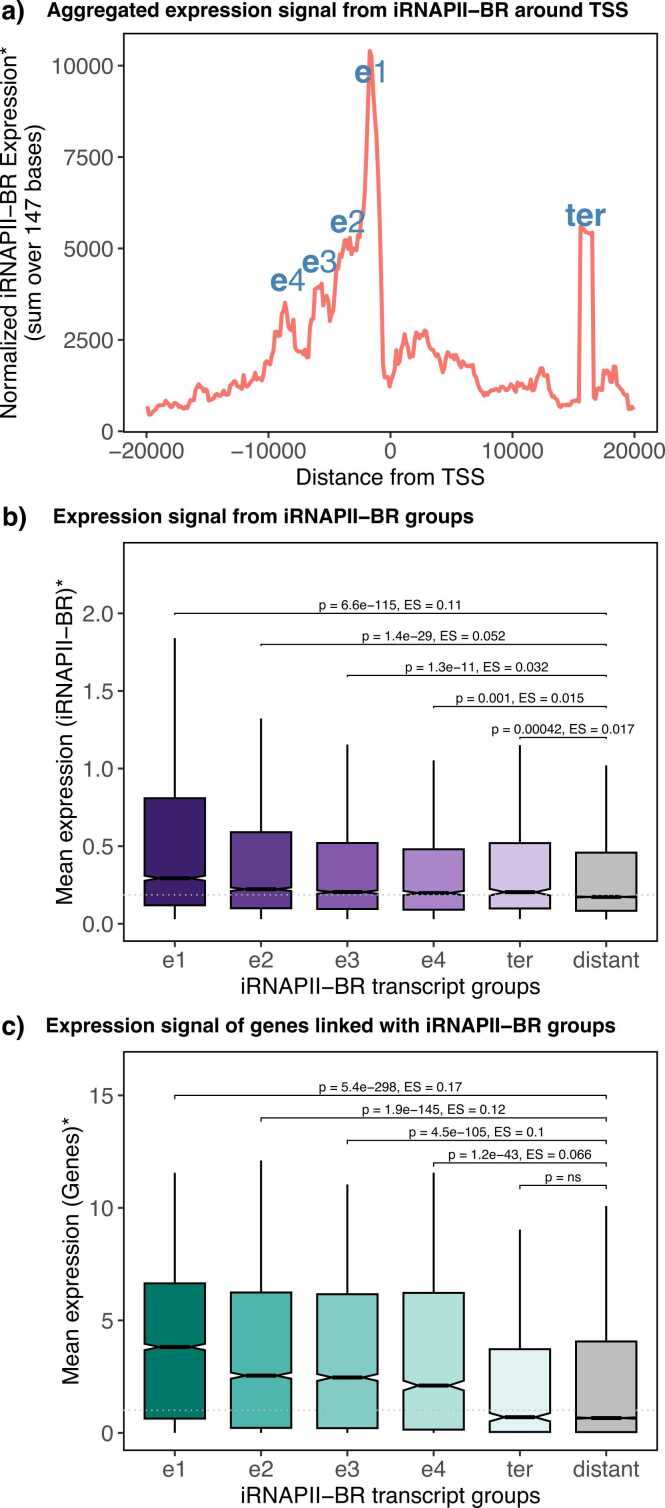
Spatial distribution of iRNAPII-BR transcript abundance according to position relative to the TSS. Panel a) shows a moving sum (147-nucleotide base window) of normalized iRNAPII-BR transcript abundance scores according to position relative to the TSS of the closest gene, revealing distinct peaks upstream and downstream of the TSS. tiRNAPII-BRs and their linked genes were classified based on these sharp peaks, which are annotated in panel a). Panel b) compares the normalized transcript abundance of the tiRNAPII-BRs within each group, whereas panel c) shows the expression of genes linked to the tiRNAPII-BRs of each group. The significance of differences between groups was assessed in Wilcoxon-tests. Asterisks indicate the mean level of expression across samples.

This profile revealed four prominent upstream peaks of tiRNAPII-BR transcripts, designated e1-e4 (Fig. 5a). The first peak (e1), located closest to the TSS had the highest transcriptional intensity, which gradually decreased from e2 to e3, and then to e4. All four peaks were positioned within 10 kb upstream from the TSSs. They corresponded to 4478, 4773, 3699, and 1942 tiRNAPII-BRs linked to 3903, 3675, 2853, and 1644 genes, respectively. Collectively, these peaks accounted for 8096 (64.01 %) of the 12,649 genes transcribed alongside their iRNAPII-BRs (Fig. 4). Genes linked to multiple tiRNAPII-BRs may overlap across peaks. The term “promoter” remains historically defined in terms of proximity to the TSS and is somewhat arbitrary [20], [4]. Given their position close to the TSS and transcriptional activity, these upstream iRNAPII-BRs may act as alternative promoters rather than distal enhancers. Furthermore, upstream tiRNAPII-BR peaks were associated with a significantly higher transcript abundance than those located further from the TSS (Fig. 5b), and the associated genes were more strongly expressed (Fig. 5c), suggesting a potential regulatory influence.

An analysis of enrichment in GO terms revealed functional differences between genes associated with the e1 to e4 groups of tiRNAPII-BRs (Figure S6), although a considerable overlap was observed between groups. The e1 group was enriched with genes corresponding to immune responses, hematopoietic differentiation, and cell adhesion. Peaks e2 and e3 were linked to T-cell activation, cell adhesion, and extracellular matrix organization, whereas the e4 group was specifically enriched in genes involved in responses to tumor necrosis factor and interleukin-1 (Figure S6).

In summary, tiRNAPII-BR transcript abundance and spatial positioning relative to the TSS were tightly coupled to the transcriptional output of neighboring genes. The clustering of highly tiRNAPII-BRs close to the TSS blurs the distinction between alternative promoters and enhancers, though these elements clearly drive blood tissue–specific gene expression across diverse biological pathways.

### 2.5 Impact of iRNAPII-BR transcription on gene expression

**Fig. 6:**
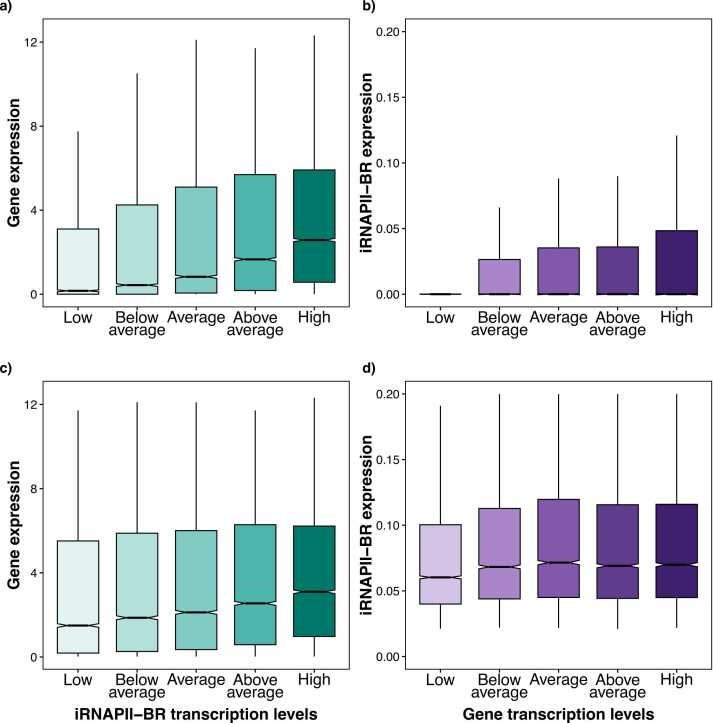
Impact of iRNAPII-BR transcript abundance on the transcription of the closest genes. Panel a) stratifies iRNAPII-BR (n = 58,259) into five quantiles based on their transcript abundance (x-axis), with the corresponding expression level for the closest gene plotted for each group (y-axis). Panel b) bins genes into five groups according to their expression levels (x-axis), with the transcript abundance of the linked iRNAPII-BRs plotted (y-axis). Panel c) and d) represent similar information for iRNAPII-BR-gene pairs (n = 46,087), in which both the iRNAPII-BR and the closest gene are transcribed.

We evaluated the influence of iRNAPII-BR transcription on gene expression by assigning iRNAPII-BRs (n = 58,259) to five categories (quantile bins) based on their transcript abundance: low, below average, average, above average, and high. We then investigated the distribution of expression for the genes associated with each group. A clear positive trend emerged, with gene expression progressively increasing with iRNAPII-BR transcript abundance across iRNAPII-BR transcript bins (Fig. 6a).

We investigated this relationship further by performing the reverse analysis, by considering the transcript abundance of iRNAPII-BRs associated with groups of genes defined according to their expression level. Contrary to the previous trend, iRNAPII-BR transcript abundance appeared to be stable across all gene expression categories (Fig. 6b). This finding suggests that higher iRNAPII-BR transcript levels may support gene activation, but that high levels of gene expression can occur independently of iRNAPII-BR activity. We also performed this analysis for pairs (n = 46,087) of genes and iRNAPII-BRs that were both transcribed (Fig. 6c,d). The results were consistent between the two comparisons.

We modeled gene expression as a function of tiRNAPII-BR transcript abundance by linear regression and calculated Spearman’s rank correlation coefficient (Figure S7). We analyzed 46,087 tiRNAPII-BR and nearest gene pairs, of which 5300 (11.5 %) displayed a significant correlation between transcript abundances, with 4507 pairs displaying a positive correlation and 793 with negative correlation (Figure S7). These tiRNAPII-BRs were associated with 3451 unique genes. A positive correlation between tiRNAPII-BRs and gene expression was expected, given the enhancer-like activity of the tiRNAPII-BRs. However, the negative correlations observed are intriguing. They may be due to transcriptional interference via several mechanisms (see Discussion).

We investigated possible regulatory mechanisms by looking for TF binding sites (TFBS) in both tiRNAPII-BRs and the promoters of the nearest transcribed genes. Specifically, we scanned 1000 bases centered on each tiRNAPII-BR and 1000 bases upstream from the TSS of the linked genes (promoters), using 2287 positional weight matrices (PWMs) [48], [60]. We found 1190 of the PWMs tested were significantly enriched in tiRNAPII-BRs, and 579 were enriched in gene promoters with p-value less than 0.05 (Dataset S4) [60]. Notably, 525 of the promoter-enriched PWMs were also enriched in tiRNAPII-BRs, suggesting a substantial overlap in regulatory potential. This overlap implies that tiRNAPII-BRs and gene promoters may be co-regulated by the same TFs. The broader and more diverse PWM enrichment in tiRNAPII-BRs is consistent with their enhancer-like properties. Enhancers are known to be hotspots for TFBS, typically characterized by the coordinated binding of multiple TFs and cofactors, often resulting in both homotypic and heterotypic complexes [53], [59].

**Fig. 7:**
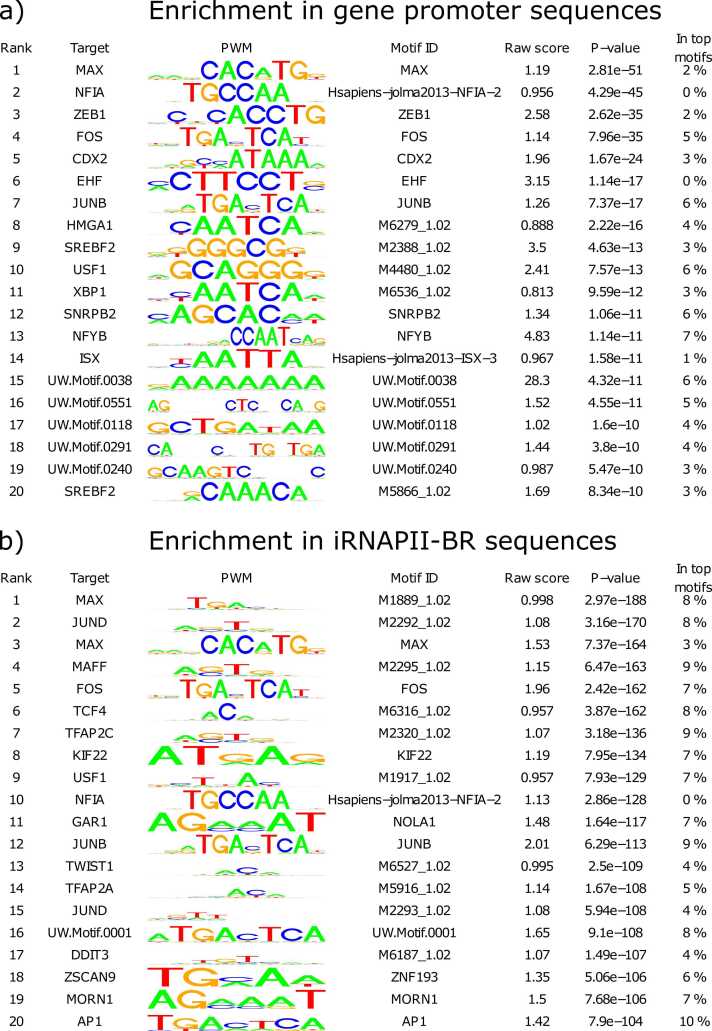
Top-ranked transcription factor binding sites displaying enrichment in the iRNAPII-BR and promoter DNA sequences for which a significant correlation between transcript abundances was detected. a) Enrichment in these sites is shown for gene promoter sequences in Panel a), and for iRNAPII-BR sequences in b).

The TFBS displaying the strongest enrichment were those for MAX, FOS, NFIA, and JUNB—factors known to be involved in transcriptional regulation, chromatin looping, and immune-related processes (Fig. 7). The MAX and JUN TF families have been linked to chromatin interactions and transcriptional activation, whereas AP-1 complex components such as FOS, JUNB, and JUND are involved in immune response, inflammation, and cellular stress [37]. Similarly, MAX is known to be involved in differentiation and proliferation, both of which are highly relevant to hematopoietic cells [29].

Collectively, these findings suggest that iRNAPII-BR transcription contributes to an upregulation of gene expression, but with a smaller subset of these regions potentially mediating downregulation. The enrichment of tiRNAPII-BRs and promoters of transcribed genes in similar TFBSs suggest their regulation by a common mechanisms.

## 3 Analysis of the differential expression of tiRNAPII-BRs and the genes closest to them in MDD

**Fig. 8:**
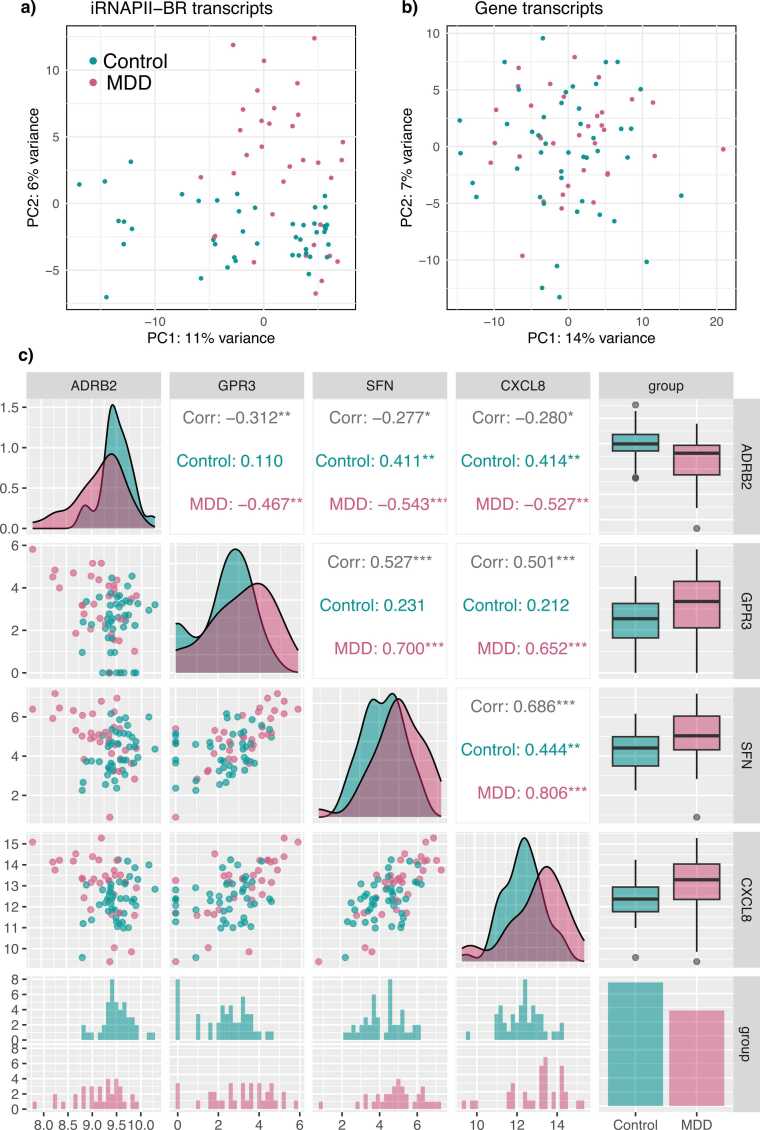
Coordinated expression of stress- and inflammation-related genes in major depressive disorder (MDD) and control samples. Panels a and b show principal component analysis of MDD and healthy control samples based on 500 highly variable tiRNAPII-BRs and genes. Panel c shows pairwise scatterplots and a correlation matrix of log₂-transformed expression levels for four genes—ADRB2, GPR3, SFN, and CXCL8—in MDD and healthy control samples. Each point represents one sample, color-coded by group. The lower panels show scatterplots with fitted trends; the diagonal panels show gene-wise expression density distributions; the upper panels show Pearson correlation coefficients. Boxplots (right) show the distribution of gene expression in control and MDD samples. Barplots show the frequency distribution for gene expression. MDD samples displayed a coordinated upregulation of GPR3, SFN, and CXCL8, and downregulation of ADRB2, reflecting a possible disruption of adrenergic and cAMP-linked stress signaling pathways.

De Langen et al. demonstrated that intergenic transcription at iRNAPII-BRs could be used as a novel biomarker for cancer diagnosis. Motivated by this case study, we performed principal component analysis on 500 highly variable features to investigate their ability to distinguish between MDD and healthy control clusters. The normalized iRNAPII-BR read counts separated MDD from controls more effectively than normalized read counts for genes (Fig. 8a,b) We also analyzed the differential expression of iRNAPII-BRs and genes between healthy controls and individuals with MDD, with DESeq2 (Table S1,S2) [43]. MA and volcano plots revealed significant differences in expression between controls and individuals with MDD for many iRNAPII-BRs and genes (Figure S8).

We identified 103 iRNAPII-BRs displaying significant differential expression in MDD (Table S3), associated with 83 unique genes. Many of these genes displayed differential expression in MDD (gene set enrichment analysis with 10,000 permutations, ranking genes according to their statistical significance when tested for differential expression in MDD: normalized enrichment score = 1.34, FDR = 0.0068). Indeed, 10 of these 83 genes linked to 16 differentially expressed iRNAPII-BRs, displayed significant differential expression in MDD with an adjusted p-value < 0.05 as the threshold for statistical significance. These genes included *ADRB2*, *CXCL8*, *GPR3*, *LINC00641*, *SFN*, and *TXNIP*, a long-non coding RNA gene (*LINC00641*), and four genes encoding for novel transcripts - ENSG00000279430, ENSG00000261468, ENSG00000261026, ENSG00000288782.

The β2-adrenergic receptor (*ADRB2*) is activated by the stress hormones epinephrine and norepinephrine, which are released during physiological and psychological stress responses [69]. Upon activation, *ADRB2* stimulates adenylate cyclase, leading to an increase in intracellular cyclic AMP (cAMP) levels. This signaling cascade protects against inflammation; for example, norepinephrine-mediated β2-adrenergic signaling has been shown to prevent hyperinflammation by inducing early IL-10 release [2]. By contrast, the *CXCL8* gene encodes interleukin-8 (IL-8), a pro-inflammatory chemokine involved in immune cell recruitment and neuroendocrine regulation. IL-8 has been linked to hypothalamic-pituitary-adrenal axis dysregulation, and high maternal IL-8 levels are associated with a higher risk of schizophrenia in offspring, highlighting the relevance of this cytokine in neurodevelopmental and neuropsychiatric disorders [8], [9]. The *GPR3* gene encodes a G-protein-coupled receptor that constitutively activates adenylate cyclase, maintaining basal cAMP levels independently of external ligands [19]. This sustained cAMP signaling may modulate stress adaptation, inflammation, or metabolic responses. *SFN* encodes stratifin (14–3–3σ), a scaffold protein involved in various signaling pathways, including protein kinase C, stress responses, and DNA damage repair [38]. *SFN* activity can intersect with cAMP-regulated pathways through shared kinase effectors and stress signaling hubs. *TXNIP* (thioredoxin-interacting protein) regulates oxidative stress and redox homeostasis, often acting as a link between cellular metabolism, inflammation, and stress-related signaling cascades.

*ADRB2* is downregulated in MDD, whereas *CXCL8*, *SFN*, and *GPR3* are upregulated (Fig. 8c). The strong co-expression of *CXCL8*, *SFN*, and *GPR3* suggests coordinated regulation of inflammatory and stress-response pathways. The low expression levels of *ADRB2* signalling probably reflect impaired adrenergic and cAMP signaling, decreasing anti-inflammatory tone and increased inflammation (e.g., via CXCL8). Simultaneous increase in *GPR3* and *SFN* expression may represent a compensatory or maladaptive response to cellular stress mediated by alternative cAMP or kinase-driven pathways.

Together, these observations support a testable neuroimmune model of depression, in which disrupted cAMP signaling acts as a molecular bridge between stress hormone dysregulation, immune activation, and oxidative stress, contributing to both peripheral and central dysfunctions in MDD.

## 4 Discussion

Our study presents a genome-wide analysis of iRNAPII-BRs and the genes to which they are linked, and their transcriptional coordination in peripheral blood. Over 82 % of iRNAPII-BRs overlapped with enhancers from the EnhancerAtlas2.0, DBNascent, ENCODE, FANTOM5, and NET-CAGE datasets, supporting their identification as enhancer-like elements. Notably, 69.3 % overlapped with DBNascent regions catalogued as bidirectionally transcribed intergenic loci, further reinforcing their classification as bidirectionally transcribed iRNAPII-BRs.

We analyzed 181,170 iRNAPII-BRs and their association with 63,150 genes to decipher their genomic and transcriptional roles. About 37.31 % (n = 23,560) of all the genes considered were linked to at least one iRNAPII-BR (Fig. 1). These genes could potentially be regulated by iRNAPII-BRs in addition to conventional promoters.

The genes linked to iRNAPII-BRs had distinctive features (Fig. 2). They were longer, had more introns, a lower promoter CpG density, occurred in low gene-density regions, and were regulated by a higher number of TFs. These genomic architectural features are closely connected to the regulatory demands and enhancer-promoter dynamics of specific cell lineages [21], [25]. For instance, neuronal genes often have larger intergenic regions and more complex enhancer-promoter interactions to accommodate their functional complexity than the genes of other lineages [30]. Genes linked to iRNAPII-BRs probably require more regulatory input due to an extended transcription time, complex processing, and spatiotemporal regulation. iRNAPII-BRs may act as transcriptional boosters, optimizing and coordinating gene transcription in a timely manner by facilitating promoter communication through chromatin looping, diffusion of transcriptional regulatory factor, the engagement of transcriptional condensates, transcription-associated domains or factories, and the maintenance of a permissive chromatin environment for sustained RNAPII activity [31], [56], [63]. By contrast, unlinked genes had simpler structure, with fewer introns, and they were located in gene-rich regions. They were regulated by fewer TFs and were probably dependent principally on their promoters or other RNA polymerases [15]. These findings indicate a regulatory dichotomy between simple, densely clustered genes and structurally complex, isolated genes. The tissue/cell type specificity of enhancers has been studied in detail. The genes linked to iRNAPII-BRs also had a low exon-to-intron ratio, potentially resulting in the generation of a larger number of isoforms to perform functions in a tissue/cell-specific manner, and their location away from gene-rich regions may facilitate the regulation of their selective expression in specific tissues/cells. It is worth noting that although the overall distributions of exon-to-intron ratios and CpG content in both gene promoters largely overlap, their median values differ, indicating moderate differences between iRNAPII-BR–linked and unlinked genes (Fig. 2b,c,d).

In peripheral blood, transcription was detected for 58,259 iRNAPII-BRs and 42,256 gene loci (Fig. 3). Transcript counts were almost perfectly positively correlated across samples. These findings suggest that bulk poly(A)-enriched RNA-seq captures a biologically meaningful subset of iRNAPII-BR transcripts. Consistent with their overlap with bidirectionally transcribed enhancers, the transcription of iRNAPII-BRs would be expected to increase — or at least reflect — the likelihood of gene activation. In total, 12,649 transcribed genes were linked to at least one tiRNAPII-BR.

Based on transcription and linkage status, we defined six gene groups with different regulatory profiles (Fig. 4). The non-transcribed genes, either unlinked to iRNAPII-BRs or linked to untranscribed iRNAPII-BRs (Groups 000 and 001), together accounted for 25.47 % (n = 16,087) of the genes considered (Fig. 4). These genes displayed an enrichment in functions and processes probably inactive in blood. Group 011 genes were linked to tiRNAPII-BRs but were not themselves transcribed. Their functions overlapped with those of Groups 000 and 001. These genes may be in a paused state or even repressed by iRNAPII-BR transcription [66].

Group 100 genes were transcribed but were not linked to any iRNAPII-BRs. This group was enriched in genes with housekeeping functions. These genes are probably regulated by promoters alone and/or are subject to RNAPII-independent gene regulation, via RNAPI and RNAPII for example [15]. Group 111 included genes cotranscribed with iRNAPII-BRs and was enriched in genes with immune and blood-specific functions. This finding supports an enhancer-like role for tiRNAPII-BRs in driving tissue-specific transcription [22], [35]. Group 101 genes were transcribed, but their linked iRNAPII-BRs were not. Surprisingly, these genes presented an enrichment in neuronal functions associated with axon and synapses. This may imply a promoter-dependent basal activation in blood with potential boosting by their iRNAPII-BRs in response to cues from the brain. The enhancer-like promoter architecture of Group 101 genes may therefore indicate cross-tissue regulation of these genes [23], [61]. Overall, genes regulated only by their promoters tended to have housekeeping roles. Genes cotranscribed with iRNAPII-BRs had tissue-specific functions. This supports the model that iRNAPII-BRs enhance context-dependent transcription [3].

We also analyzed the spatial distribution of transcripts from tiRNAPII-BRs with respect to the location of the tiRNAPII-BR relative to the TSS (Fig. 5). We observed four prominent peaks located within 10 kb upstream of TSSs, with transcript abundance progressively decreasing with increasing distance from the TSS. The transcript abundance of each tiRNAPII-BR closely mirrored the level of expression of the gene closest to it. Given their spatial organization and proximity to promoters, these regions appear more like alternative or upstream promoters rather than classical distal enhancers. Our results therefore support the emerging view that enhancer-like regions can initiate transcription, and may acting as alternative promoters [14], [5], [52], [57], [7].

Genes linked to iRNAPII-BRs with a high transcript abundance were more strongly expressed (Fig. 6). However, grouping genes by expression level revealed that high levels of gene expression could occur independently of iRNAPII-BRs. The transcript levels of tiRNAPII-BRs were mostly correlated with the level of expression of the nearest gene, consistent with their enhancer-like functions in gene activation. However, some intriguing negative correlations were also observed. The chromatin in these regions is probably in an open configuration as both the gene and the iRNAPII-BR were transcribed. The observed negative correlations may therefore be due to transcriptional interference. If the tiRNAPII-BR is too close to the promoter or on the same strand, the RNAPII molecules may collide or occlude each other. Some genes may encode factors that repress their own enhancers. The tiRNAPII-BR may link to multiple promoters, potentially leading to dynamic promoter competition.

The tiRNAPII-BRs and the promoters of their co-expressed closest genes showed enrichment for the same TFBS (Fig. 7). These shared motifs included those recognized by key regulators of chromatin organization and immune responses in blood. tiRNAPII-BRs contained approximately twice as many TFBS as their paired promoters, consistent with the well-known role of enhancers as TFBS-rich regulatory hotspots. Notably, 91 % of promoter TFBS were also present in the corresponding tiRNAPII-BRs, supporting both classical and emerging models of enhancer–promoter communication. In the classical view, shared TFBS can facilitate enhancer–promoter interactions through DNA looping, potentially stabilized by homodimerizing transcription factors bound at both elements [32]. However, recent high-resolution 3D genome studies challenge the universality of stable looping and instead propose alternative mechanisms. For example, iRNAPII-BRs could be alternative or genuine promoters considering the enrichment for the same TFs and previously reported unifying properties [14], [5], [52], [57], [7]. Karr et al. propose a model that CREs act as local biochemical reactors, generating tunable gradients of modified or activated TFs that diffuse to nearby promoters [31]. The near-complete TFBS overlap between tiRNAPII-BRs and their nearest promoters aligns well with this model. Furthermore, the active transcription of iRNAPII-BRs implies recruitment of extensive transcriptional machinery to these elements, which may itself contribute to local diffusion gradients that extend from the enhancer toward adjacent promoter regions [31].

Interestingly, tiRNAPII-BRs with expression levels varying considerably between samples separated MDD patients from healthy controls more effectively than genes with highly variable expression (Fig. 8a,b), indicating that bulk RNA-Seq data can be used beyond their intended scope. A small subset of 103 tiRNAPII-BRs, linked to 83 genes displayed expression changes in MDD. Ten of these genes displayed significant differential expression. Notably, the dysregulation of *ADRB2*, *CXCL8*, *SFN*, and *GPR3* may be relevant to MDD (Fig. 8c). In particular, *ADRB2*-mediated anti-inflammatory signaling appears to be suppressed in MDD [2], whereas the pro-inflammatory pathways associated with *CXCL8*, *SFN*, and *GPR3* are upregulated. Interestingly, *CXCL8* gene shows differential expression in second-generation antipsychotic drug induced metabolic syndrome and impaired immune functions in schizophrenia patients [62]. Consistently, these genes are directly or indirectly connected to cAMP signaling and the hypothalamic–pituitary–adrenal axis [38]. Disruptions of cAMP signaling may serve as a molecular bridge linking stress hormone imbalance, immune activation, and oxidative stress, thereby contributing to both the peripheral and central dysfunctions observed in MDD. Independent experimental validation is required to confirm these findings in the context of MDD pathology and their potential for use as biomarkers for MDD.

Altogether, our findings show that integrating iRNAPII-BR analyses with routine gene-expression profiling can substantially enhance our understanding of transcriptional regulation and disease biology. Notably, the same RNA-Seq dataset can yield an additional regulatory layer, enabling multi-omics or multiview analyses without extra experimental cost [12], [46], [67]. Transcription from iRNAPII-BRs provides high-resolution information on RNAPII occupancy and potential TFBSs, which can be integrated with resources such as scBlood—a comprehensive single-cell accessible-chromatin atlas of blood cells across 30 tissues and 21 diseases—to illuminate regulatory mechanisms in hematopoiesis and blood-related disorders [72]. These data can further support the identification of active regulatory elements through integration with large-scale transcriptional regulatory networks [49], [73], prediction of active enhancers and promoters and their combinations [12], and the analysis of regulatory-region mutations [71]. Such integrations would enable identification of early regulatory perturbations, discovery of disease-specific enhancer–promoter rewiring, and mapping of cell-state transitions driven by altered RNAPII dynamics. Ultimately, these synergies allow a more mechanistic interpretation of how transcriptional dysregulation shapes disease onset and progression, yielding clearer biomarkers, therapeutic targets, and functional hypotheses for experimental validation.

## 5 Conclusion

Our genome-wide analysis indicates that iRNAPII-BRs are bidirectionally transcribed, enhancer-like regions that may contribute to the spatial and temporal regulation of gene expression in blood. These regions preferentially associate with structurally complex genes characterized by long gene bodies, low-CpG promoters, low exon-to-intron ratios, location outside gene-dense regions, and regulation by a larger number of transcription factors. This organization may support selective and context-dependent transcriptional control. Some of these associations showed modest effect sizes, and further work using datasets such as ENCODE, Tfit, and dREG will be important to more clearly define the features distinguishing iRNAPII-BR–linked from unlinked genes. Transcription of iRNAPII-BRs was generally associated with higher gene expression but does not appear universally required, consistent with an auxiliary, enhancer-like role. Their cotranscription and frequent proximity to TSSs suggest that some may act as alternative promoters rather than distal enhancers. The shared TFBS signatures between iRNAPII-BRs and their nearest promoters may permit either classical DNA-looping interactions or, alternatively, support emerging TF activity gradient model where CREs serve as local hubs assembling transcriptional machinery, generating diffusible gradient from enhancers to promoters. The strong overlap in TFBS and the presence of transcriptional machinery at tiRNAPII-BRs align more with the transcription factor gradient model. Overall, these observations point to a modulatory role for iRNAPII-BRs in fine-tuning gene expression and contributing to tissue-specific isoform diversity. Although the RNA-seq data used here were enriched for polyadenylated transcripts—whereas many enhancer RNAs are non-polyadenylated—and although bulk RNA-seq captures intergenic transcription only indirectly due to RNA stability differences, the detected iRNAPII-BR signals remain biologically meaningful within this context. Integrating iRNAPII-BR profiling with standard transcriptome analyses may therefore provide additional layers of regulatory insight from the same RNA-seq data and improve our understanding of context-dependent transcriptional regulation, including its potential relevance in conditions such as MDD.

## 6 Material and methods

### 6.1 RNA-Sequencing data processing and analyses

The raw sequencing data in FASTQ format were pre-processed with fastp to remove adapter sequences and low-quality reads [13]. Cleaned reads were then aligned with the human genome reference (GRCh38) with the Align function from the Rsubread package [40], generating BAM files that mapped reads to genomic coordinates. Mapping statistics were generated for each sample, and two samples with less than 50 % mapped reads were excluded to maintain data quality. Read counts were then computed with the featureCounts function from Rsubread [41]. We quantified transcripts within 1 kb windows centered around 181,547 RNAPII binding regions and for annotated genes [35]. We therefore produced two count matrices—one for genes and one for iRNAPII-BRs—for 85 samples.

We ensured data consistency by performing correlation analyses separately for gene expression and iRNAPII-BR data. Samples deviating by more than two standard deviations from the mean value were considered outliers and were excluded. We also retained only genes and iRNAPII-BRs with at least one read in three or more samples to minimize noise.

DESeq2 was used for differential expression analysis and principal compoenent analysis [43]. Differential expression between healthy control and MDD samples was analyzed separately for genes and iRNAPII-BRs. Differences with an adjusted p-value < 0.05 were considered significant.

### 6.2 Annotation of iRNAPII-BRs, gene characteristics, and functional enrichment analysis

Gene annotation data were obtained from the Homo_sapiens.GRCh38.111.gtf.gz file from the ENSEMBL server [27]. Gene lengths were calculated based on the transcription start and end sites of each gene. The exon-to-intron ratio was determined by subtracting the total exon length from the gene length to obtain intron length and then calculating the ratio of exon to intron length. Gene density around each gene was calculated by counting the number of genes within 100,000 nucleotides flanking the gene and normalizing this value by dividing by 200,000 nucleotides. CG dinucleotide frequency was calculated separately for genes and for the 1000 nucleotides upstream from the TSS. Information on the TFs regulating each gene was obtained from a previous study [49]. RNAPII binding sites were annotated with the annotatePeakInBatch function from the ChIPpeakAnno Bioconductor package [75], with gene annotation information extracted from the ENSEMBL GTF file. The TF binding motifs were identified with the PWMEnrich Bioconductor package [48], [60]. Briefly, mean affinity was calculated over each of the iRNAPII-BRs and promoter sequences and compared with that of length-matched sequences from the genomic background. GO term enrichment analysis was performed with clusterProfiler [68]. Where necessary, gene name synonyms were converted with theorg.Hs.eg.db annotation package [10].

## CRediT authorship contribution statement

**Vijaykumar Yogesh Muley:** Conceptualization, Methodology, Formal analysis, Investigation, Writing - Original Draft, Writing - Review & Editing. **Andrée Delahaye-Duriez:** Conceptualization, Supervision, Investigation, Funding acquisition, Project administration, Resources, Writing - Review & Editing"

## Conflict of interest

The authors declare no competing interests.

## Data Availability

This study is based on data obtained from previously published studies, as cited in the text. MDD RNA-seq data are publicly available via the Gene Expression Omnibus accession GSE251786 (https://www.ncbi.nlm.nih.gov/geo/query/acc.cgi?acc=GSE251786). All other relevant data are included in the article as supplementary material.

## Appendix A Supplementary material

**Dataset S1:** Dataset provides genomic annotation of intergenic RNAPII-bound regions (iRNAPII-BRs). For each region, the table lists the unique identifier from the original study (de Langen et al., 2024, PMID: 37868033), chromosomal coordinates (chromosome, start, end), and the distance from the region midpoint to the transcription start site of the nearest Ensembl-annotated gene. Gene information includes Ensembl ID, genomic coordinates, and strand orientation. The dataset also reports binary overlap (1 = overlapping, 0 = non-overlapping) between each iRNAPII-BR and enhancer annotations obtained from DBNascent, FANTOM5, ENCODE, NET-CAGE, and EnhancerAtlas2.0, enabling integrative analysis of regulatory activity and enhancer associations.

**Dataset S2:** SummarizedExperiment object containing RNA-seq read counts mapped to intergenic RNAPII-bound regions (iRNAPII-BRs) in human peripheral blood samples from healthy donors and MDD patients. The dataset integrates region-level count matrices (assays), genomic coordinates for each iRNAPII-BR (rowData), and detailed sample metadata (colData).

**Dataset S3:** SummarizedExperiment object containing gene-level RNA-seq read counts from human peripheral blood samples obtained from healthy individuals and patients diagnosed with major depressive disorder (MDD). The dataset includes raw read counts (assays), comprehensive gene annotations (rowData), and curated sample metadata (colData).

**Dataset S4:** Dataset reports transcription factor binding sites (TFBS) detected within intergenic RNAPII-bound regions (iRNAPII-BRs) and within the promoters (1 kb upstream of the TSS) of their nearest Ensembl-annotated genes. For each iRNAPII-BR, the table includes the original region identifier (de Langen et al., 2024, PMID: 37868033), chromosomal coordinates (chromosome, start, end), and the list of significant TFBS identified within the region. The distance from the region midpoint to the gene transcription start site is provided for reference, along with the Ensembl gene ID. TFBS detected within the corresponding gene promoter are listed, and the final column reports the intersection of TFBS present in both the iRNAPII-BR and its nearest gene promoter. This dataset enables comparative analysis of shared regulatory motifs between regulatory regions and gene promoters.

**Figure S1:** The distribution of Encode and DBNascent CREs overlapping with iRNAPII-BRs, categorized according to their known classifications. The bar lengths represent the proportion of overlapping CREs in each category relative to the total number of overlapping sites. This analysis highlights the types of regulatory region most frequently coinciding with iRNAPII-BRs, suggesting potential enrichment patterns or functional associations. Panel a) shows a functional classification of CREs from Encode annotation data. Panel b) shows CREs obtained from the DBNascent database, predicted with Tfit and dREG techniques.

**Figure S2:** Inverse relationship between presence of iRNAPII-BRs and local gene density. a) We investigated whether iRNAPII-BRs were associated with gene density, for each gene, by defining a TSS-centered ±100 kb window and counting overlapping iRNAPII-BRs (y-axis). Gene density was measured as the number of genes within a given ±100 kb window (x-axis). b) The plot shows gene density binned into 20 density classes on the x-axis, and the mean number of iRNAPII-BRs corresponding to the genes in these classes on the y-axis.

**Figure S3:** Gene ontology term enrichment for genes linked and unlinked to iRNAPII-BRs.

**Figure S4:** Characteristic features of protein-coding genes with (in purple n=8,395) and without associated iRNAPII-BR (in gray n=11,641). The p-value (p) and effect size (ES) for comparisons between two groups were determined in Wilcoxon rank-sum tests.

**Figure S5:** Sample outlier selection and filtering. Panel a shows the percentage of reads mapping to the genome in each sample (order by depth). Panels b and c show standard deviation plots of inter-sample correlations based on the iRNAPII-BR count matrix and the gene count matrix, respectively.

**Figure S6:** Gene ontology term enrichment for genes associated with transcribed iRNAPII-BRs clustered around the TSS in four peaks, e1-e4, a downstream peak ter, with the remaining iRNAPII-BRs considered distal.

**Figure S7:** Distribution of Spearman correlation coefficients for the relationship between iRNAPII-BR transcript abundance and the expression level of the linked genes. Panel a shows all correlations, and panel b shows only significant correlations.

**Figure S8:** Differential expression of genes and iRNAPII-BR transcripts in MDD and control samples. MA and volcano plots are shown for genes (a) and iRNAPII-BR transcripts (b).

**Table S1:** Differential expression of iRNAPII-BRs in MDD versus Control samples.

**Table S2:** Differential expression of genes in MDD versus Control samples.

**Table S3:** iRNAPII-BRs with significant expression differences in MDD compared to control samples.

## Footnotes

Appendix A Supplementary data associated with this article can be found in the online version at doi:10.1016/j.csbj.2025.11.060.
