# Supplementary figures and images for: Transcriptional coordination between intergenic RNA polymerase II-bound regions and nearby genes reveals functional specialization and disease associations in peripheral blood
Source: Comput Struct Biotechnol J. 2025 Nov 28;27:5523–36. doi: 10.1016/j.csbj.2025.11.060 (PMC12722025; doi:10.1016/j.csbj.2025.11.060)

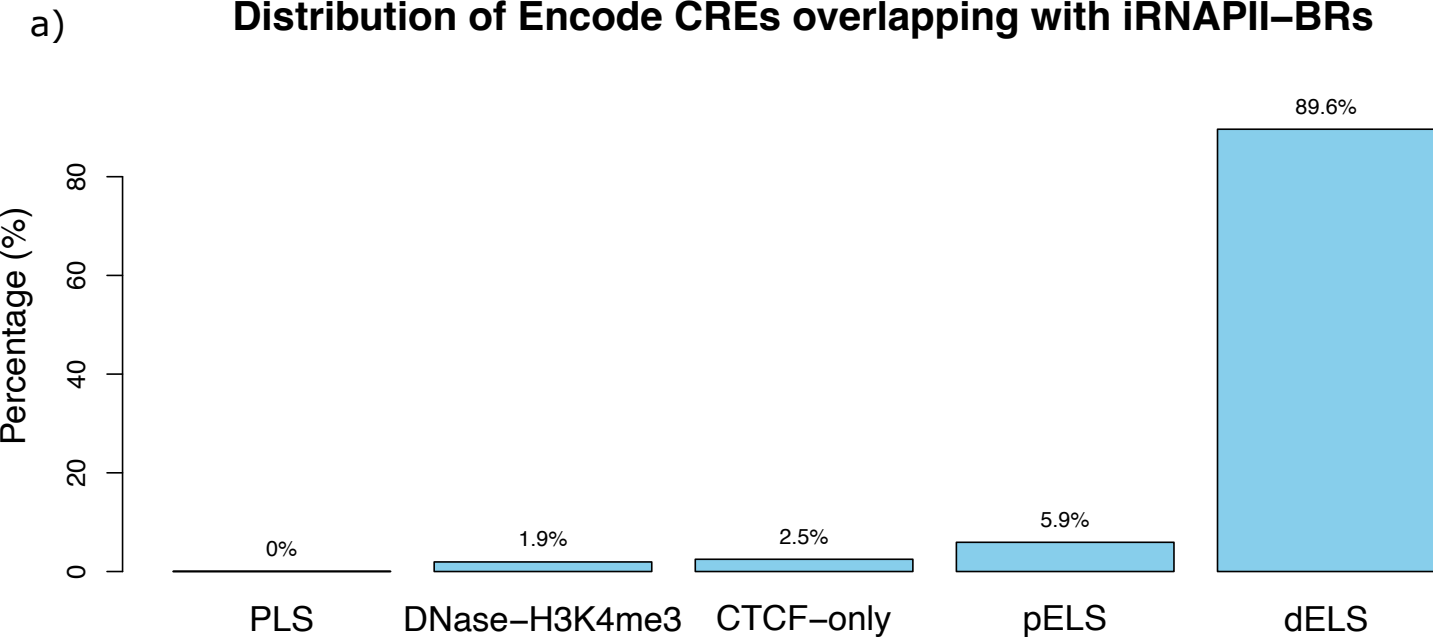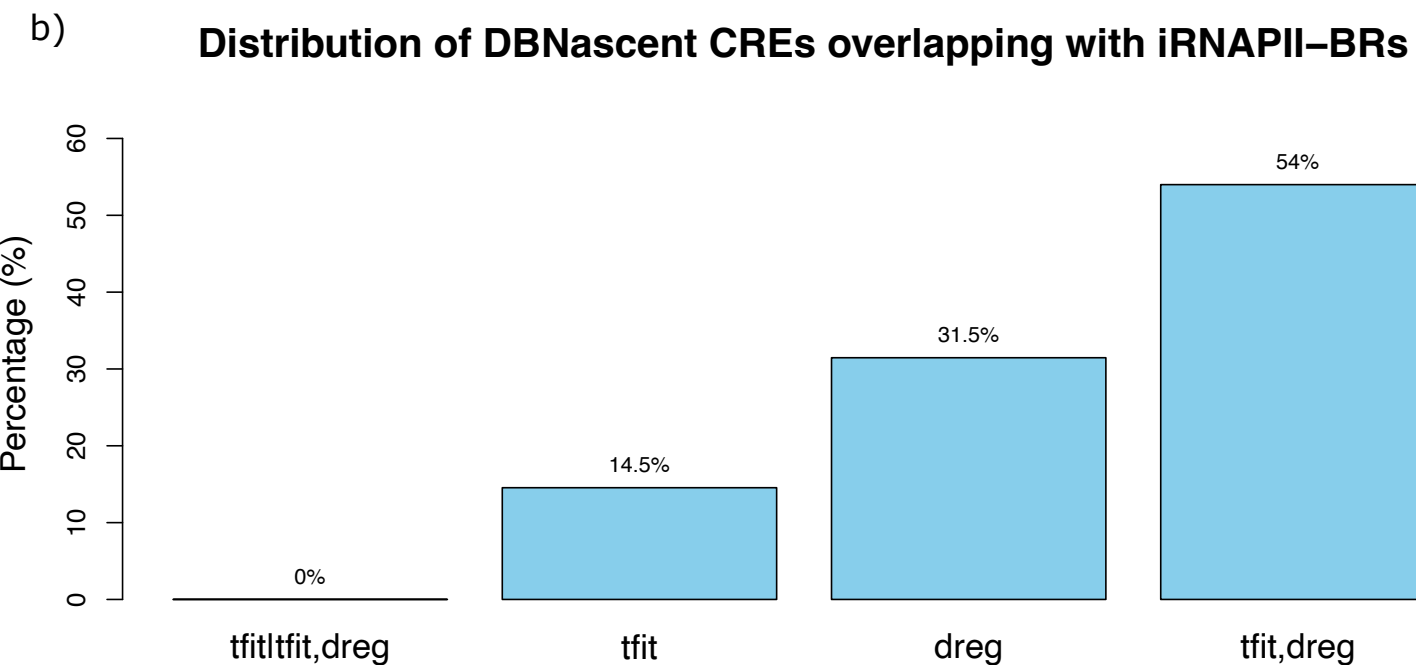

Supplement: Supplementary file 5 — Figure S1: The distribution of Encode and DBNascent CREs overlapping with iRNAPII-BRs, categorized according to their known classifications. The bar lengths represent the proportion of overlapping CREs in each category relative to the total number of overlapping sites. This analysis highlights the types of regulatory region most frequently coinciding with iRNAPII-BRs, suggesting potential enrichment patterns or functional associations. Panel a) shows a functional classification of CREs from Encode annotation data. Panel b) shows CREs obtained from the DBNascent database, predicted with Tfit and dREG techniques. [file mmc5.pdf]

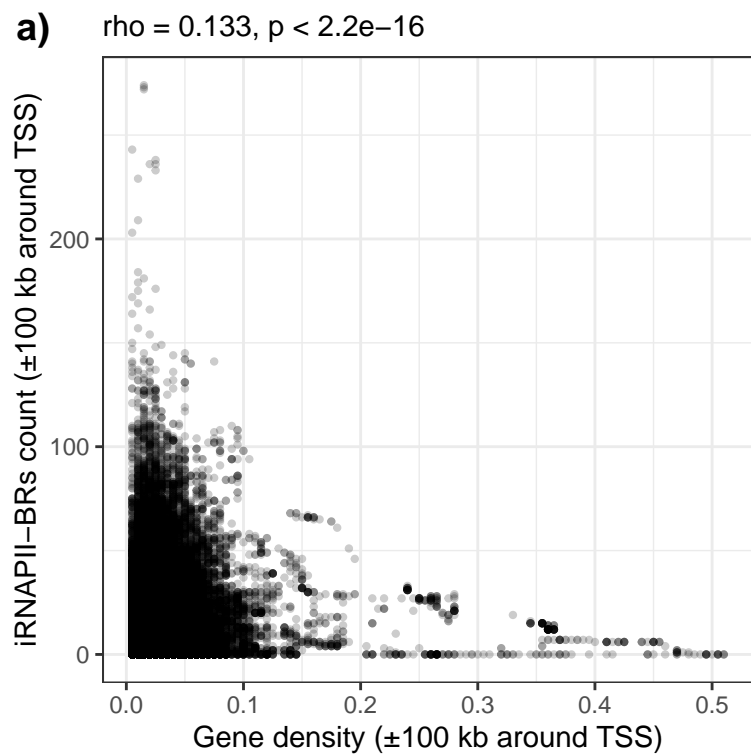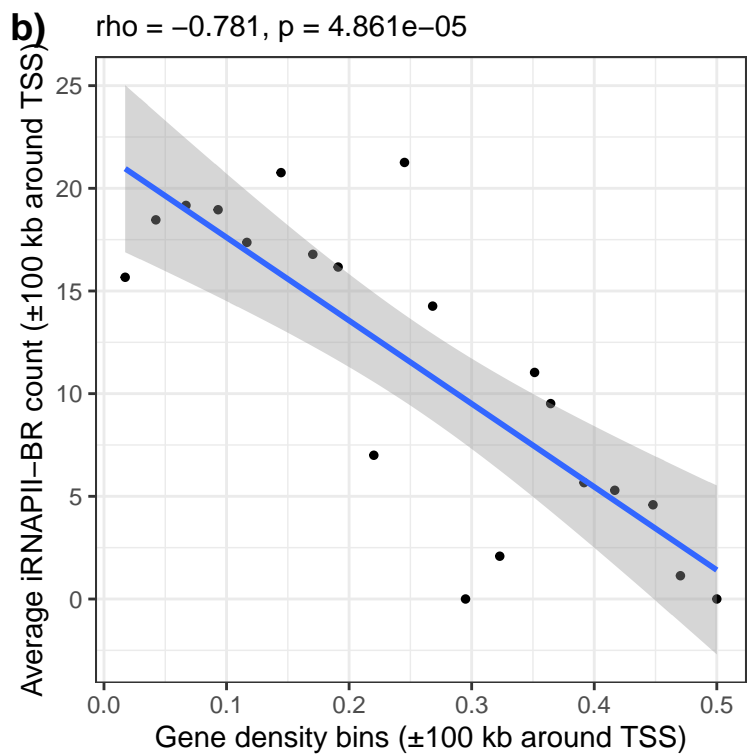

Supplement: Supplementary file 6 — Figure S2: Inverse relationship between presence of iRNAPII-BRs and local gene density. a) We investigated whether iRNAPII-BRs were associated with gene density, for each gene, by defining a TSS-centered ±100 kb window and counting overlapping iRNAPII-BRs (y-axis). Gene density was measured as the number of genes within a given ±100 kb window (x-axis). b) The plot shows gene density binned into 20 density classes on the x-axis, and the mean number of iRNAPII-BRs corresponding to the genes in these classes on the y-axis. [file mmc6.pdf]

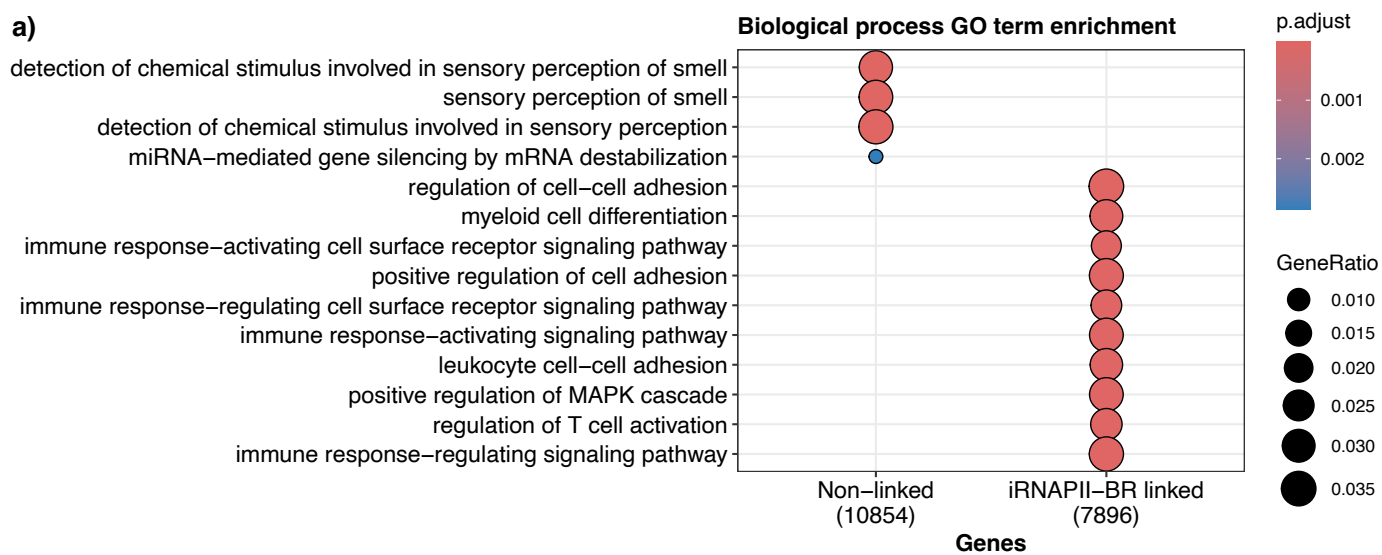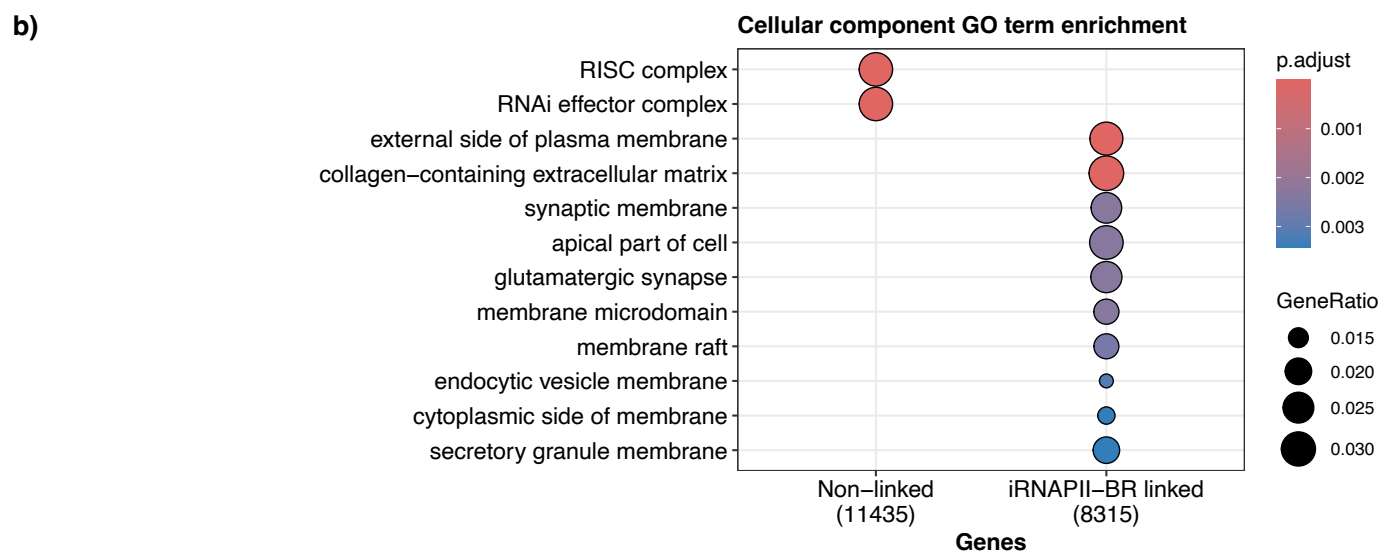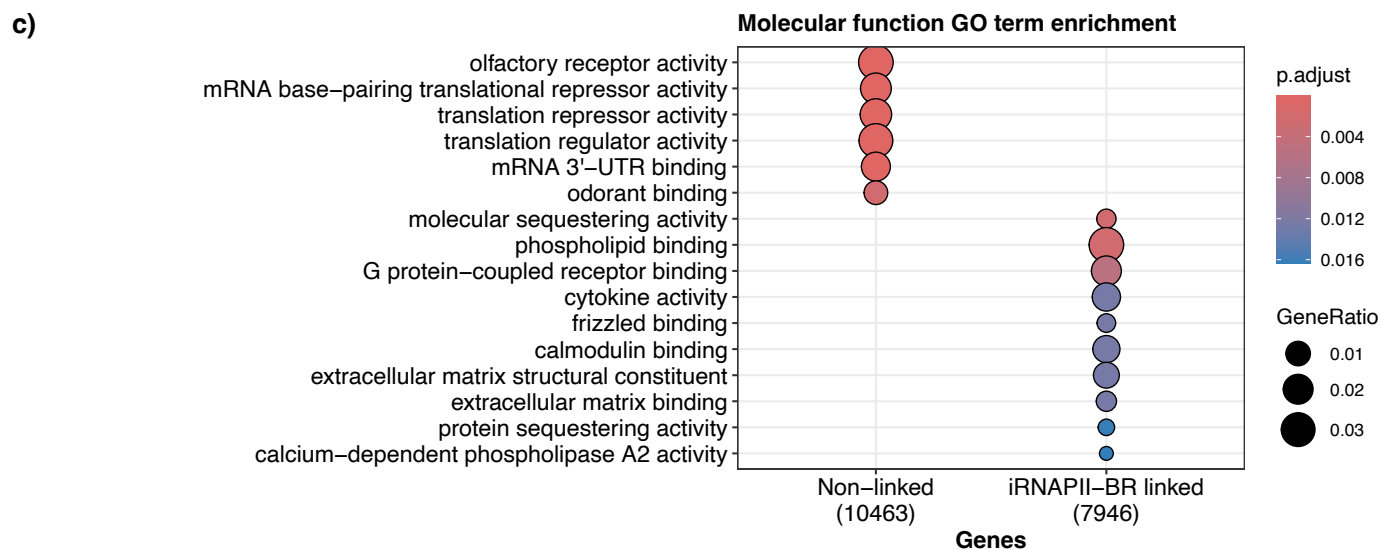

Supplement: Supplementary file 7 — Figure S3: Gene ontology term enrichment for genes linked and unlinked to iRNAPII-BRs. [file mmc7.pdf]

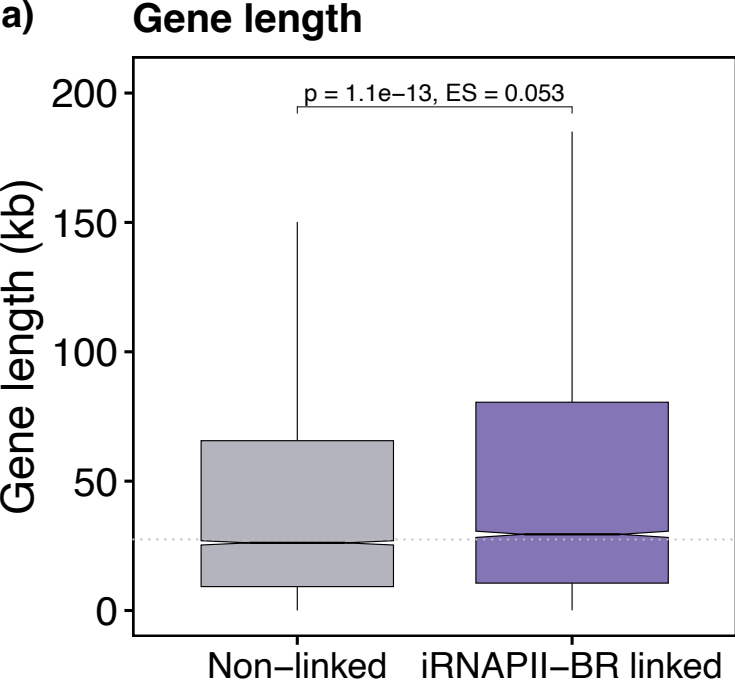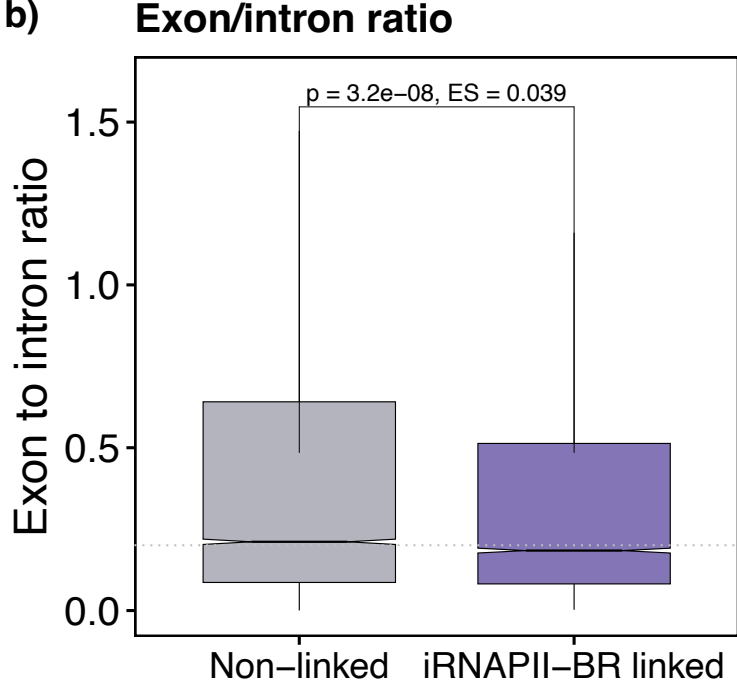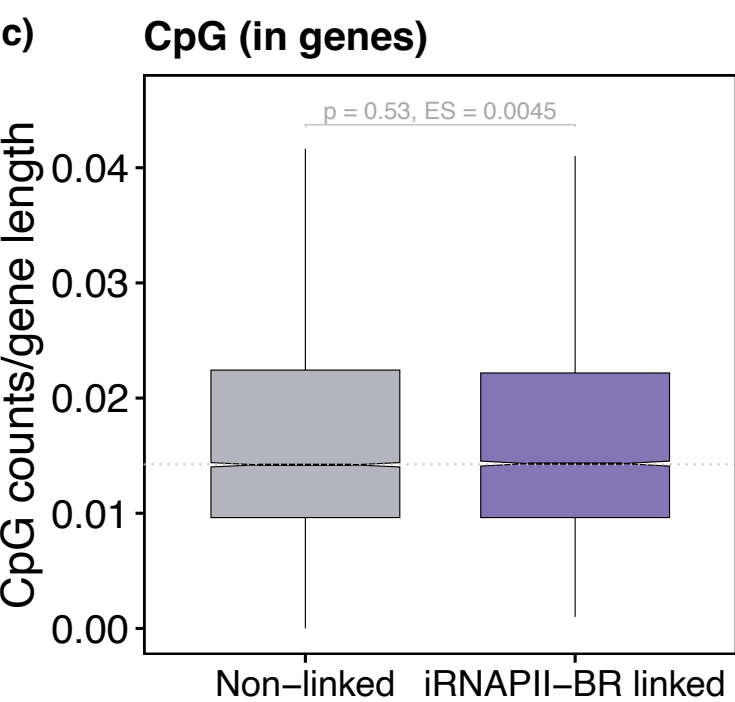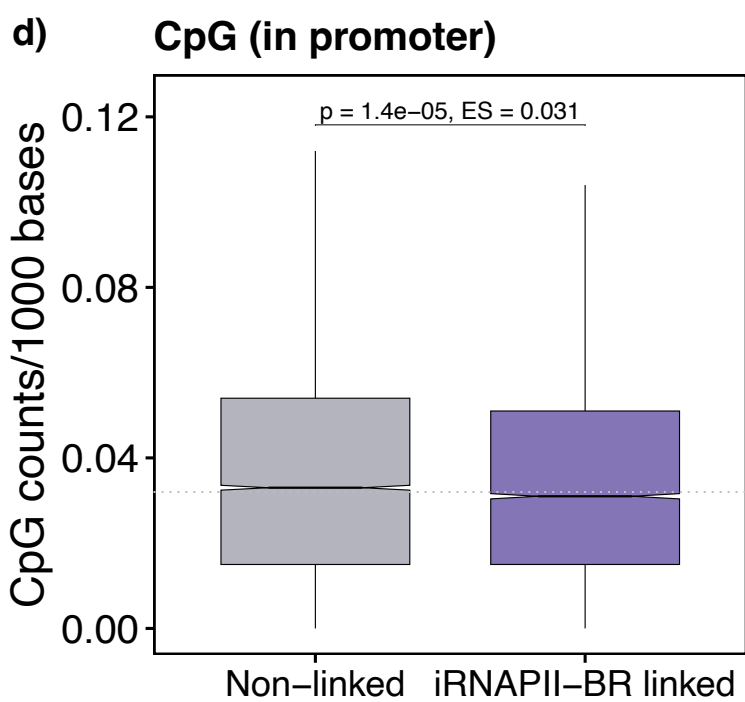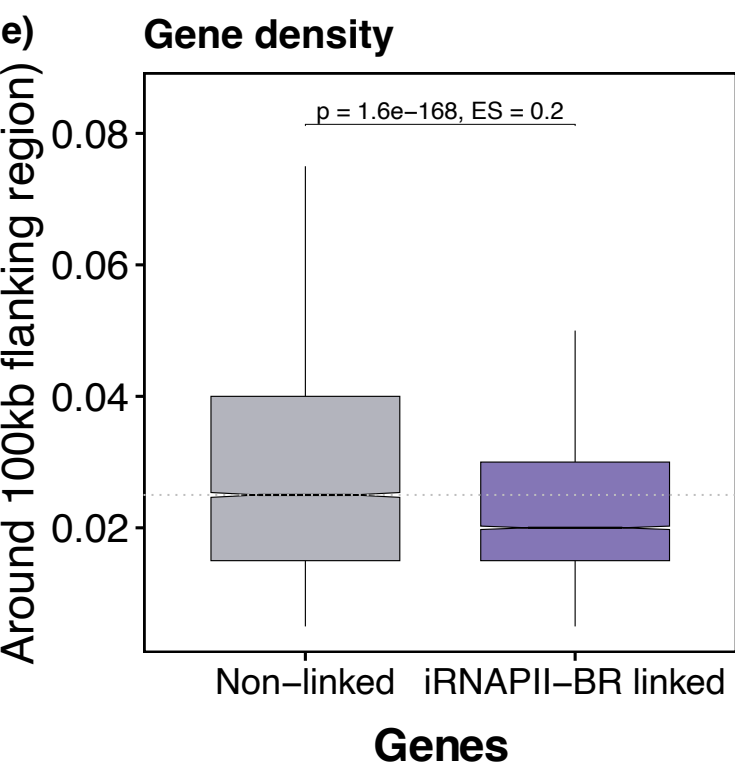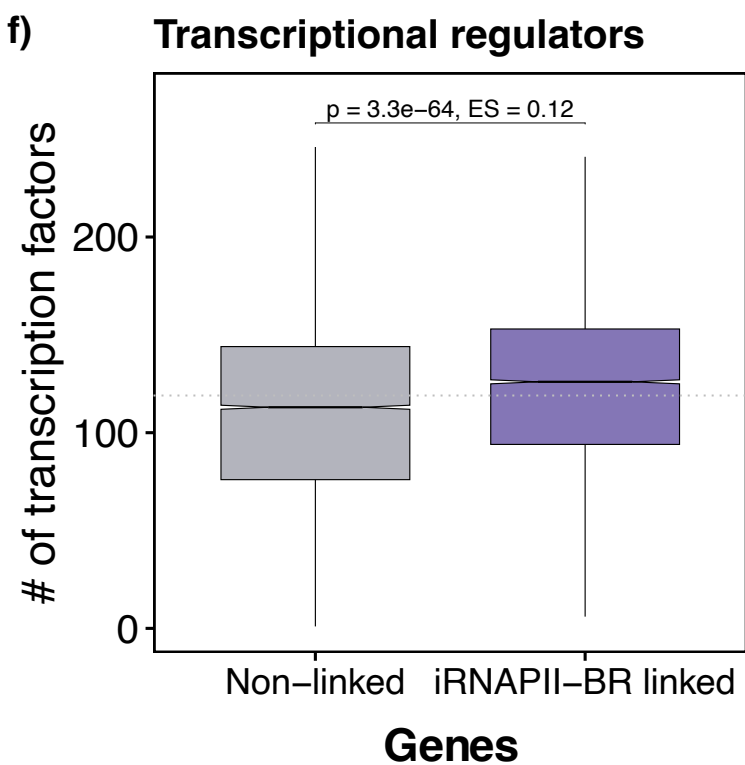

Supplement: Supplementary file 8 — Figure S4: Characteristic features of protein-coding genes with (in purple n=8,395) and without associated iRNAPII-BR (in gray n=11,641). The p-value (p) and effect size (ES) for comparisons between two groups were determined in Wilcoxon rank-sum tests. [file mmc8.pdf]

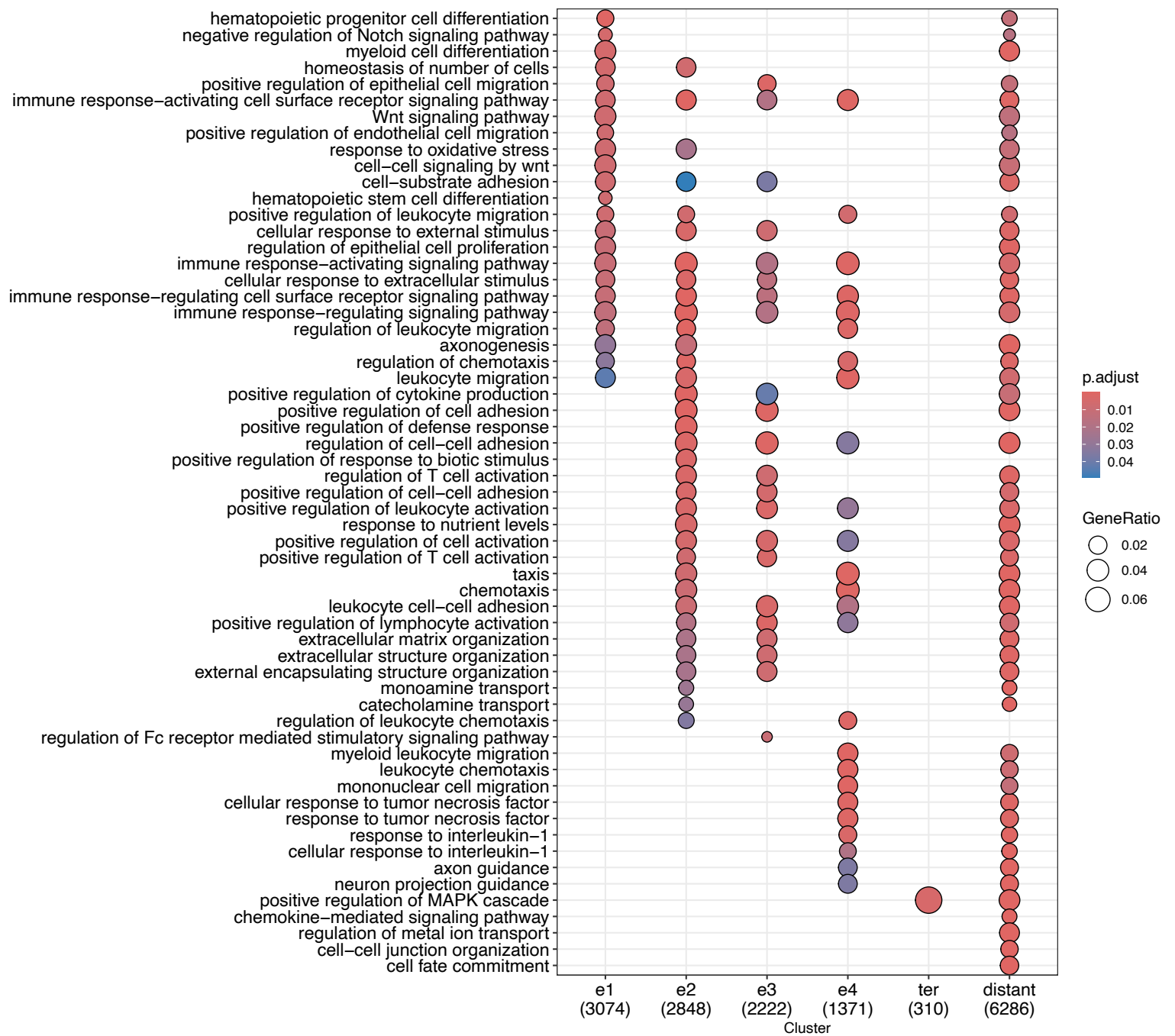

Supplement: Supplementary file 10 — Figure S6: Gene ontology term enrichment for genes associated with transcribed iRNAPII-BRs clustered around the TSS in four peaks, e1-e4, a downstream peak ter, with the remaining iRNAPII-BRs considered distal. [file mmc10.pdf]

**a)** Expression correlations (iRNAPII-BRs and nearest genes)

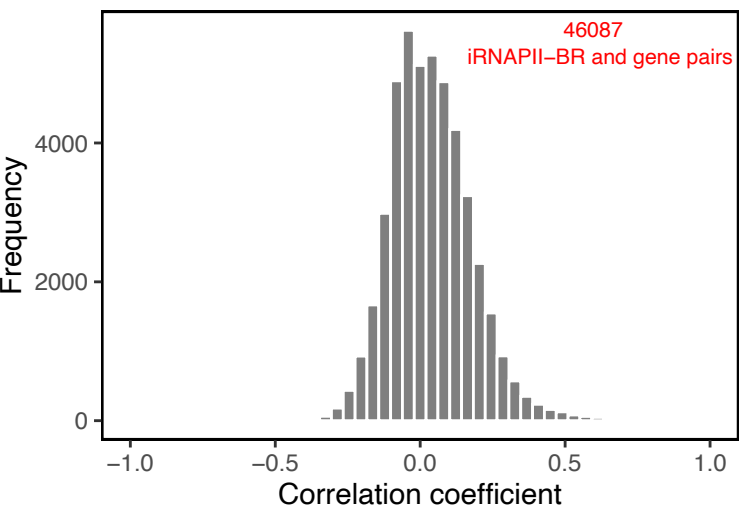

**b)**

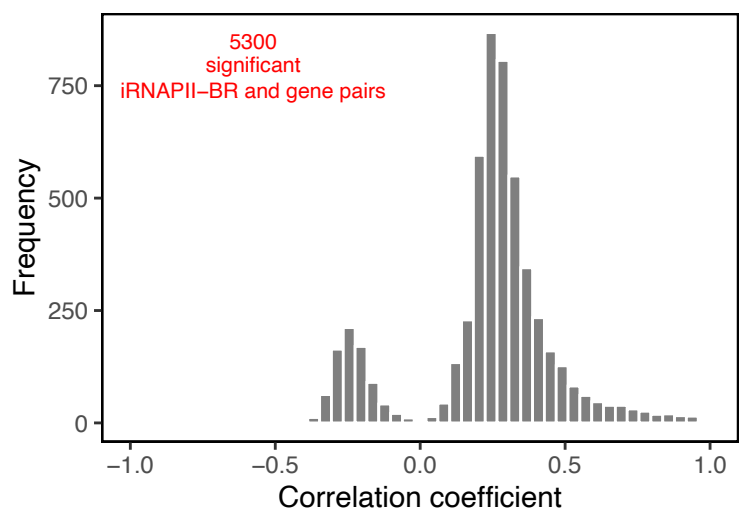

Supplement: Supplementary file 11 — Figure S7: Distribution of Spearman correlation coefficients for the relationship between iRNAPII-BR transcript abundance and the expression level of the linked genes. Panel a shows all correlations, and panel b shows only significant correlations. [file mmc11.pdf]

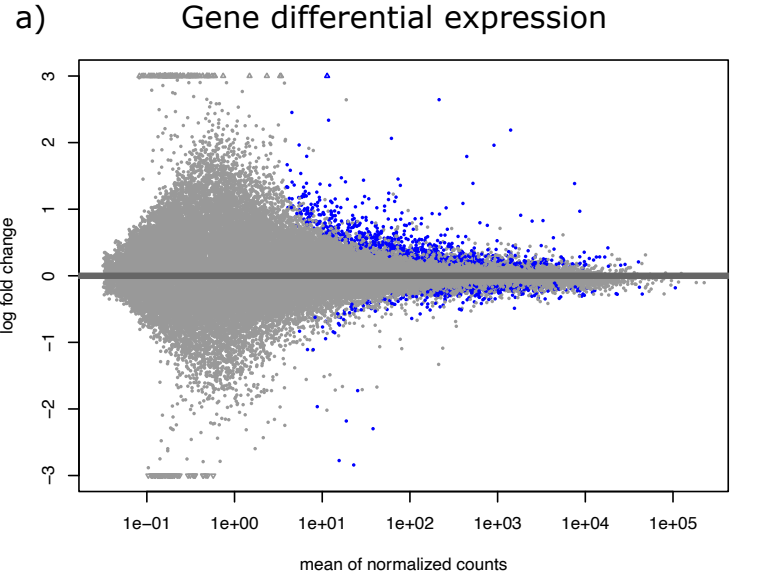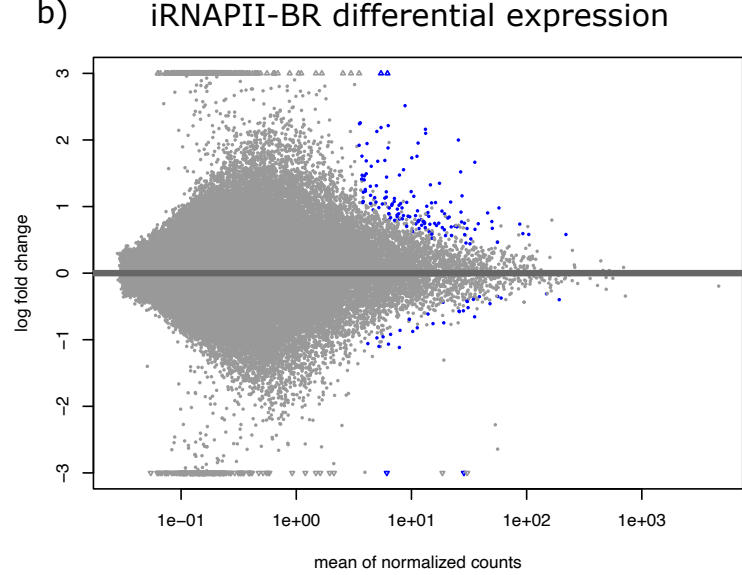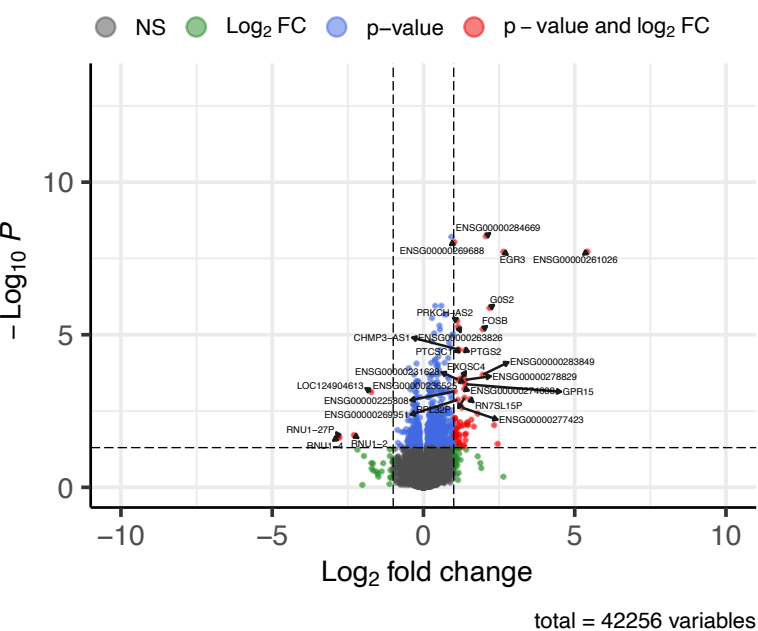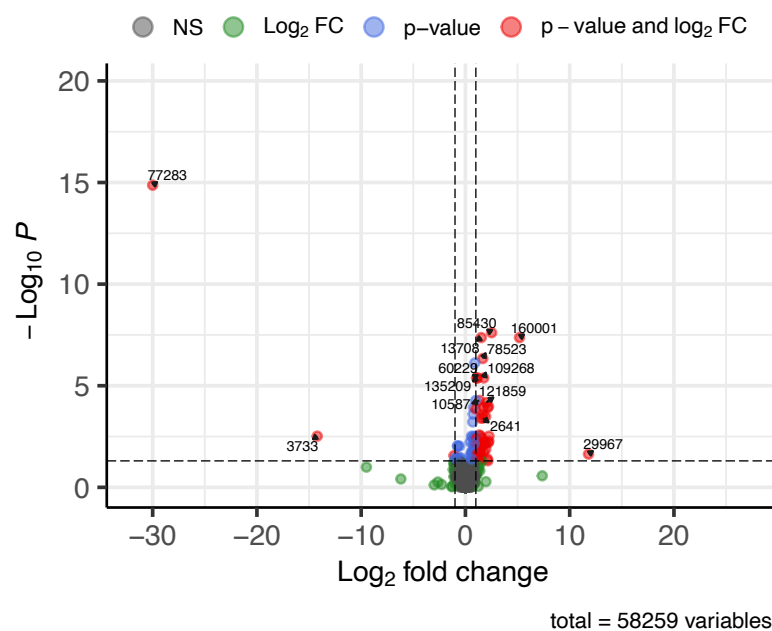

Supplement: Supplementary file 12 — Figure S8: Differential expression of genes and iRNAPII-BR transcripts in MDD and control samples. MA and volcano plots are shown for genes (a) and iRNAPII-BR transcripts (b). [file mmc12.pdf]
