# Supplementary material for: Transcriptional coordination between intergenic RNA polymerase II-bound regions and nearby genes reveals functional specialization and disease associations in peripheral blood
Source: Comput Struct Biotechnol J. 2025 Nov 28;27:5523–36. doi: 10.1016/j.csbj.2025.11.060 (PMC12722025; doi:10.1016/j.csbj.2025.11.060)
Supplement: Supplementary file 9 — Figure S5: Sample outlier selection and filtering. Panel a shows the percentage of reads mapping to the genome in each sample (order by depth). Panels b and c show standard deviation plots of inter-sample correlations based on the iRNAPII-BR count matrix and the gene count matrix, respectively. [file mmc9.pdf]

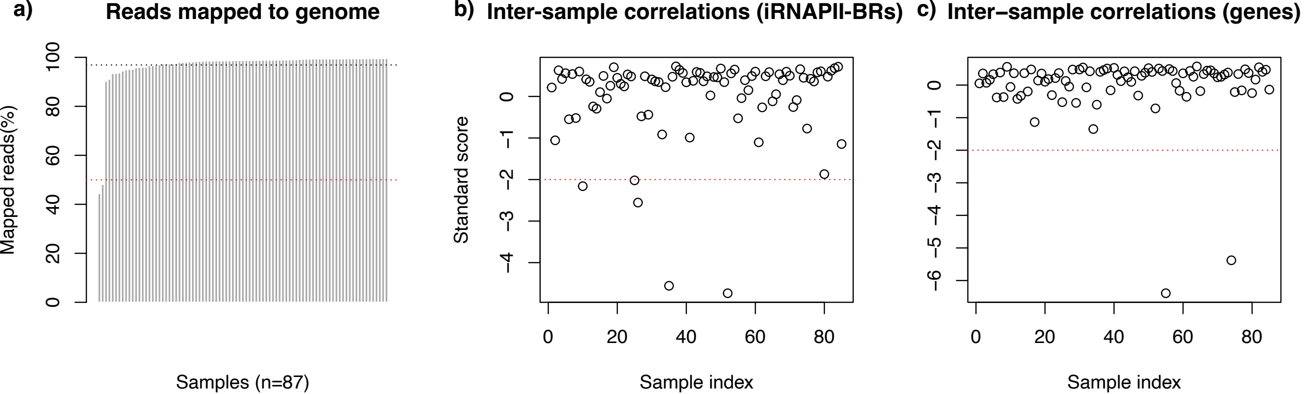
a)
Reads mapped to genome
Mapped reads(%)
0
20
40
60
80
100
Samples (n=87)
b)
Inter-sample correlations (iRNAPII-BRs)
Standard score
0
−1
−2
−3
−4
0
20
40
60
80
Sample index
c)
Inter−sample correlations (genes)
0
−1
−2
−3
−4
−5
−6
0
20
40
60
80
Sample index
